# Supplementary material for: Spatially bound functional heterogeneity drives modular organization in β-cell networks
Source: Biophys J. 2025 Aug 5;124(18):3008–22. doi: 10.1016/j.bpj.2025.07.043 (PMC12709238; doi:10.1016/j.bpj.2025.07.043)
Supplement: Document S1. Figures S1–S4 [file mmc1.pdf]

Biophysical Journal, Volume *124*

Supplemental information

Spatially bound functional heterogeneity drives modular organization in β-cell networks

Maja Duh, Marko Šterk, Lidija Križančić Bombek, Patrick E. MacDonald, Andraž Stožer, and Marko Gosak

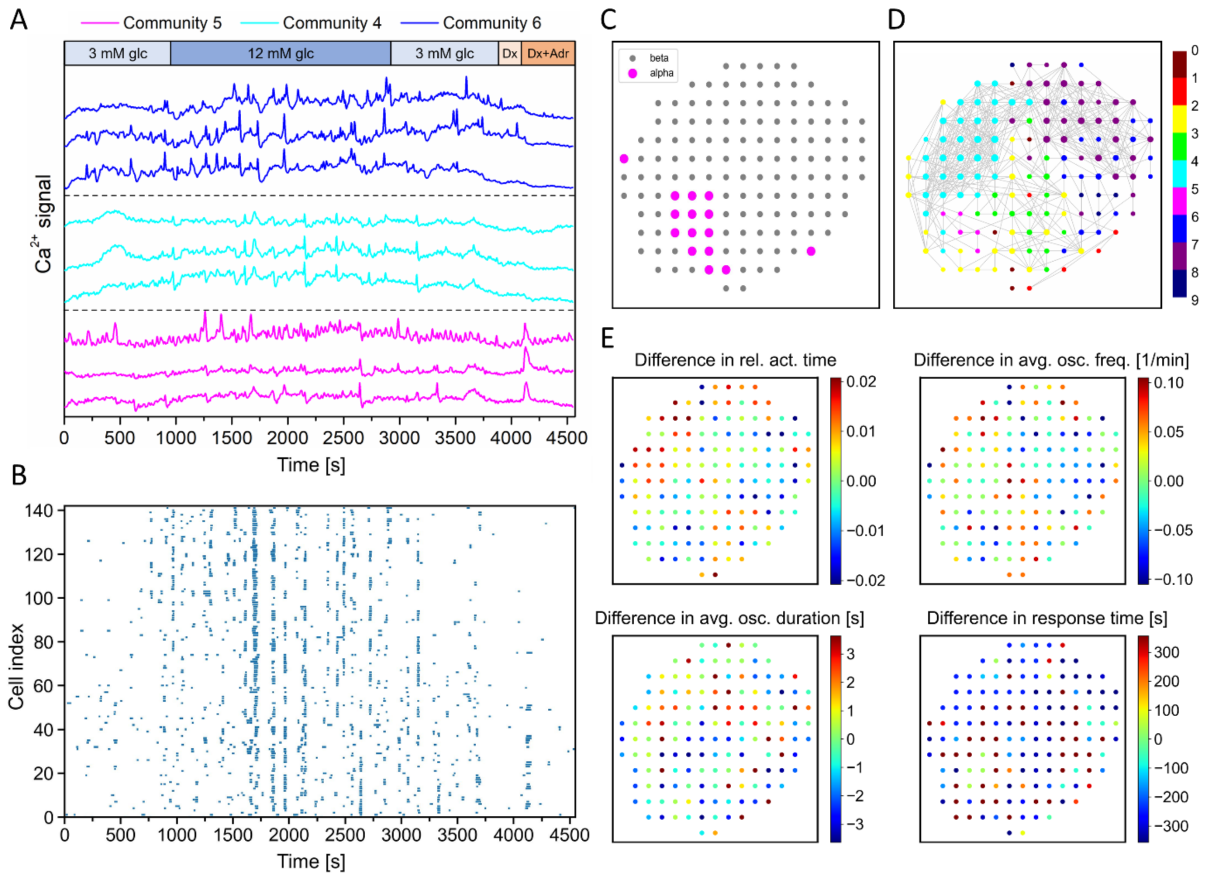

Figure S1. Multicellular activity and community structure of alpha–beta cell networks in a representative human islet. (A) Representative Ca^{2+} traces from cells belonging to three different communities within the human islet, indicated by distinct colors (trace colors correspond to the community colors in the network shown in panel D). The first three traces (magenta, Community 5) originate from a community composed of alpha cells, while the remaining traces (cyan and blue, Communities 4 and 6) correspond to beta-cell communities. Cells were first incubated in 3 mM glucose, followed by stimulation with 12 mM glucose for 30 minutes, and then returned to 3 mM glucose. After 15 minutes, the islet was briefly exposed to 3 mM glucose supplemented with 100 µM Diazoxide, followed by stimulation with 3 mM glucose + 100 µM Diazoxide + 10 µM Adrenaline. (B) Raster plot showing binarized Ca^{2+} activity of all recorded cells across the stimulation protocol. (C) Spatial distribution of alpha (magenta) and beta (gray) cells within the representative human islet. (D) Functional network of the human islet, constructed using an average node degree of $k_{avg} \approx 8$. Node colors represent distinct communities as defined by modularity analysis (see color bar). (E) Alpha and beta cells color-coded according to deviations from the islet-wide average for different Ca^{2+} signaling parameters: relative active time, average oscillation frequency, average oscillation duration, and response time (as indicated by the respective color scales).

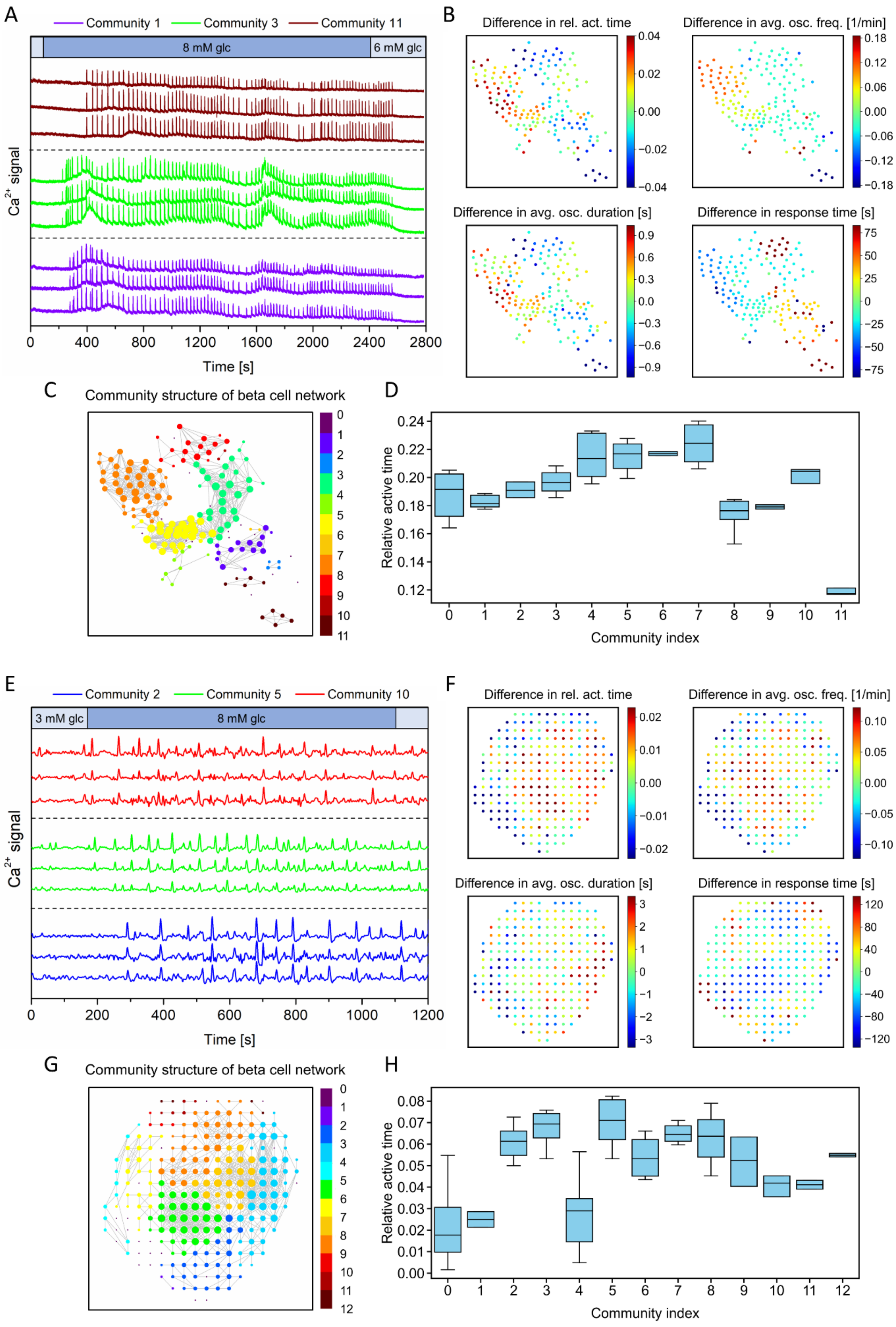
A
Community 1
Community 3
Community 11
8 mM glc
6 mM glc
Ca2+ signal
Time [s]
B
Difference in rel. act. time
Difference in avg. osc. freq. [1/min]
Difference in avg. osc. duration [s]
Difference in response time [s]
C
Community structure of beta cell network
D
Relative active time
Community index
E
Community 2
Community 5
Community 10
3 mM glc
8 mM glc
Ca2+ signal
Time [s]
F
Difference in rel. act. time
Difference in avg. osc. freq. [1/min]
Difference in avg. osc. duration [s]
Difference in response time [s]
G
Community structure of beta cell network
H
Relative active time
Community index

Figure S2: Multicellular activity and community structure of beta cell networks in representative mouse (panels A-D) and human (panels E-H) islets. (A) and (E) Three Ca^{2+} signals of cells from three different communities within the representative mouse (A) and human (E) islet, as indicated by different colors (colors on signals correspond to communities in networks (C) and (G)). (B) and (F) Beta cells within mouse (B) and human (F) islet color-coded in accordance with the variations in different Ca^{2+} signalling parameters, expressed as the difference of the value in a given cell from the whole islet average, for: the relative active time, average oscillation frequency, average oscillation duration and response time, as specified by the color bars. (C) and (G) Functional islet networks for mouse (C) and human (G) islet with an average network node degree ($k_{avg} \approx 8$). Colors of cells indicate different communities, as specified by the color bar. (D) and (H) Distribution of relative active time values in different communities of the corresponding mouse (D) and human (H) islet network. Boxes of individual communities determine the 25th and 75th percentile, whiskers denote the 10th and 90th percentile, and the lines within boxes indicate the median values.

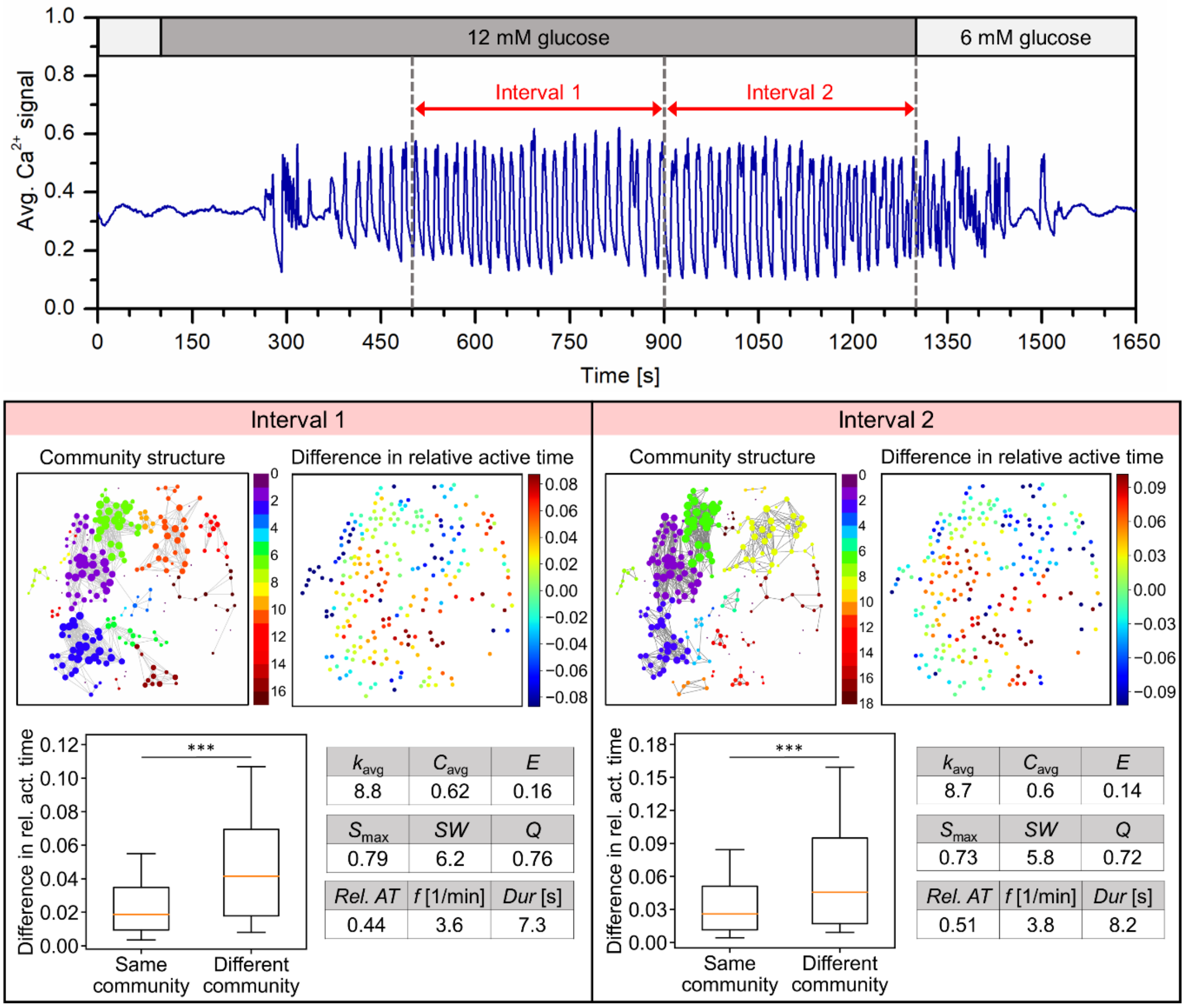

Figure S3: Temporal persistency of spatial distribution of functional heterogeneity in beta cell networks in a mouse islet. The upper panel shows the average Ca^{2+} signal from all cells within the islet, stimulated with 12 mM glucose, with designated time intervals used for the analysis. The lower panel is divided into Interval 1 and Interval 2, which were used for further analysis. For each time interval, functional beta cell networks were constructed. In these networks, colored dots represent the physical locations of cells within the islet, grey lines indicate functional connections, and the colors of the cells denote different communities. Next to this, cells within the islet are color-coded according to their differences in relative active time, as specified by the color bar. Individual values are defined as the difference between the active time of a single cell and the average active time of the entire islet. Additionally, the differences in active time within the same and different communities are shown for both intervals. Tables on the right summarize the calculated network parameters: average node degree (k_{avg}), average clustering coefficient (C_{avg}), global efficiency (*E)*, relative size of the largest component (S_{max}), small-world coefficient (*SW*), and modularity (*Q*). It also includes key cell activity parameters: relative active time (*AT*), average oscillation frequency (*f*), and average oscillation duration (*Dur*). Statistical significance: $*p < 0.05$, $**p < 0.01$, $***p < 0.001$; n.s., not significant.

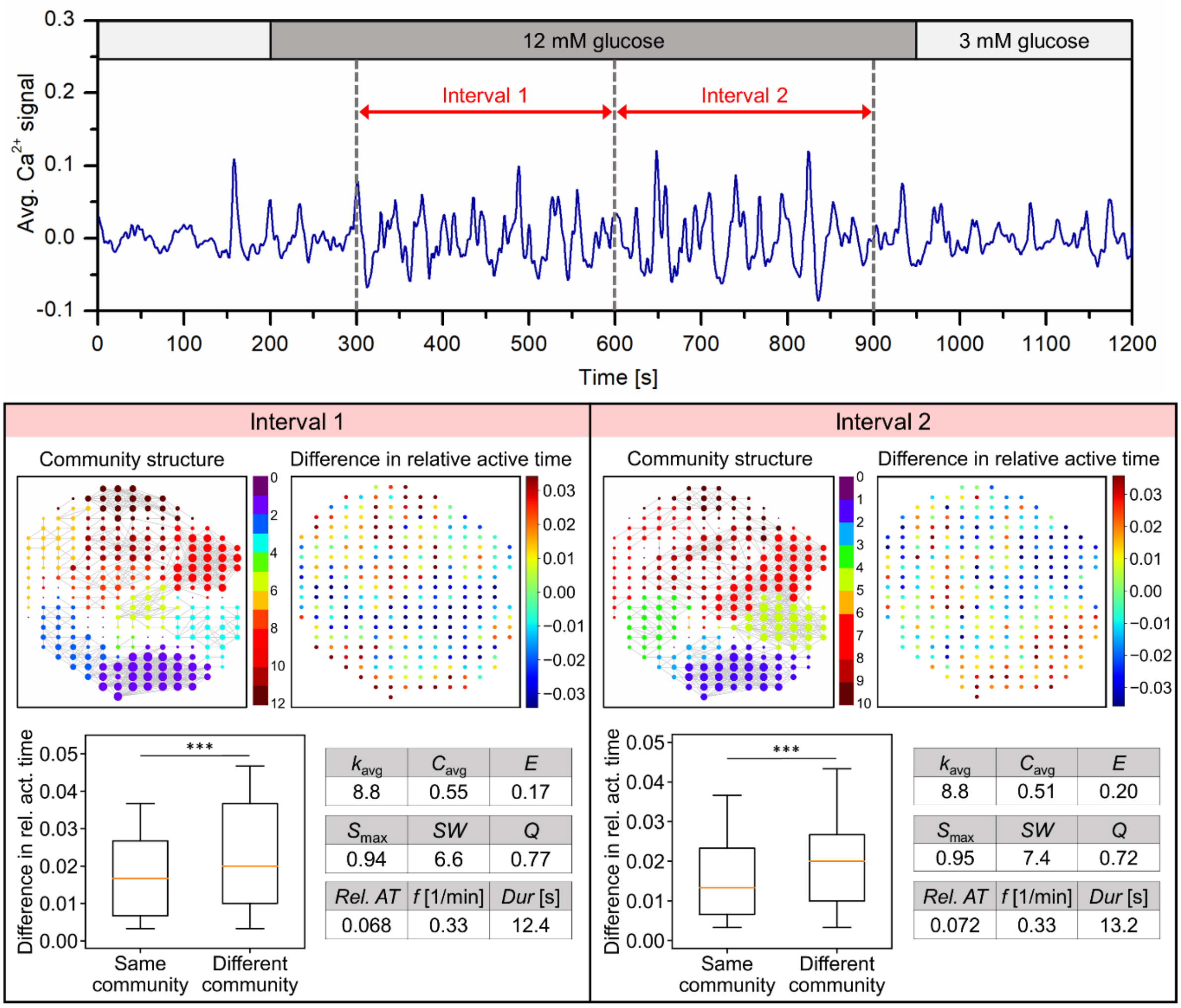

Figure S4: Temporal persistency of spatial distribution of functional heterogeneity in beta cell networks in a human islet. The upper panel shows the average Ca^{2+} signal from all cells within the islet, stimulated with 12 mM glucose, with designated time intervals used for network analysis. The lower panel is divided into Interval 1 and Interval 2, which were used for further analysis. For each time interval, functional beta cell networks were constructed. In these networks, colored dots represent the physical locations of islet subregions within the islet, grey lines indicate functional connections, and the colors of the subregions denote different communities. Next to this, islet subregions within the islet are color-coded according to their differences in relative active time, as specified by the color bar. Individual values are defined as the difference between the active time of a single islet subregion and the average active time of the entire islet. Additionally, the differences in active time within the same and different communities are shown for both intervals. Tables on the right summarize the calculated network parameters: average node degree (k_{avg}), average clustering coefficient (C_{avg}), global efficiency (*E)*, relative size of the largest component (S_{max}), small-world coefficient (*SW*), and modularity (*Q*). It also includes key cell activity parameters: relative active time (*AT*), average oscillation frequency (*f*), and average oscillation duration (*Dur*). Statistical significance: $*p < 0.05$, $**p < 0.01$, $***p < 0.001$; n.s., not significant.
