# Supplementary material for: Spatially bound functional heterogeneity drives modular organization in β-cell networks
Source: Biophys J. 2025 Aug 5;124(18):3008–22. doi: 10.1016/j.bpj.2025.07.043 (PMC12709238; doi:10.1016/j.bpj.2025.07.043)
Supplement: Document S2. Article plus supporting material [file mmc4.pdf]

Biophysical Journal
Article

Biophysical Society

Spatially bound functional heterogeneity drives modular organization in β-cell networks

Maja Duh,[1,2,3] Marko Šterk,[1,3,4] Lidija Križančić Bombek,[1] Patrick E. MacDonald,[5] Andraž Stožer,[1,*] and Marko Gosak[1,2,3,*]
[1]Institute of Physiology, Faculty of Medicine, University of Maribor, Maribor, Slovenia; [2]Department of Physics, Faculty of Natural Sciences and Mathematics, University of Maribor, Maribor, Slovenia; [3]Alma Mater Europaea University, Maribor, Slovenia; [4]Institute of Information Science, Maribor, Slovenia; and [5]Department of Pharmacology and Alberta Diabetes Institute, University of Alberta, Edmonton, AB, Canada

ABSTRACT Coordinated responses of pancreatic β-cell networks to changes in extracellular nutrient concentrations are a well-established phenomenon with significant implications for insulin secretion. This study investigates the organization and spatiotemporal activity patterns of collective β-cell dynamics and their relationship to functional network structures. Our findings highlight that functional heterogeneity among β-cells is reflected in oscillatory Ca^{2+} activity that varies within the islet, with spatially adjacent β-cells often exhibiting similar signaling characteristics. We observe a progressive response of β-cells to increasing glucose levels, where they become activated in smaller clusters as glucose levels transition from substimulatory to stimulatory. Furthermore, we show that functional β-cell networks are highly modular, with community structures strongly influenced by the cells' spatial positions and aligning partially with Ca^{2+} activity clusters and, more significantly, with clusters of simultaneous activations in response to glucose increases, highlighting the interrelated nature of these phenomena and the presence of general organizational principles that hold true in both mouse and human islets. We also observe that specific subpopulations, i.e., first responder, wave initiator, and hub cells, are principally distributed across different communities. In mouse islets, their presence is more dependent on their location within the islet, while in human islets, it is more influenced by the activity of the given subregion.

SIGNIFICANCE Understanding how β-cells coordinate their responses to fluctuating nutrient levels is vital for unraveling the mechanisms of insulin secretion. Our findings reveal that β-cells with similar Ca^{2+} signaling characteristics cluster together, forming highly modular networks that include specialized subpopulations such as hub, wave initiator, and first responder cells. This spatially organized heterogeneity may support robust, adaptable, and stimulus-dependent tuning of collective dynamics—features difficult to achieve in homogeneous systems. Importantly, this organizational principle is conserved in both mouse and human islets. These findings offer new perspectives on islet architecture and function, informing future efforts in computational modeling, therapeutic modulation of islet signaling, and the engineering of stem cell-derived β-cell assemblies.

INTRODUCTION

Multicellular structures interpret external cues through the collective actions of cellular populations. Given the dynamic and often intense nature of changes in the extracellular environment, various strategies have evolved to ensure robust responses within cellular networks. One such strategy is inherent cell-to-cell variability. While cells may share the same differentiation state, even isogenic cells can exhibit substantial variation in the expression or activity of key metabolic or signal transduction pathways, resulting in functionally diverse behaviors. This variability gives rise to distinct subpopulations—clusters of cells with shared properties—that enable more precise recognition and tailored responses to environmental changes and stimulation (1–3). This is true for the pancreatic islets of Langerhans, which are ellipsoidal clusters of endocrine cells within the pancreatic parenchyma, playing a crucial role in metabolic homeostasis by secreting important hormones such as insulin from islet β-cells (4,5). β-Cells function by sensing nutrients, primarily glucose, and coupling that to cellular depolarization, action potential firing, intracellular Ca^{2+}

Submitted May 8, 2025, and accepted for publication July 31, 2025.

*Correspondence: andraz.stozer@um.si or marko.gosak@um.si

Editor: Richard Bertram.
https://doi.org/10.1016/j.bpj.2025.07.043

© 2025 The Author(s). Published by Elsevier Inc. on behalf of Biophysical Society.
This is an open access article under the CC BY license (http://creativecommons.org/licenses/by/4.0/).

responses, and, ultimately, the exocytotic release of insulin (6). These events occur in a characteristic oscillatory manner (7,8), the impairment of which is observable in type 2 diabetes (9). Heterogeneity of β-cells has been known for decades, including variation in metabolism, electrical activity, intracellular Ca^{2+} responses, and insulin secretion (10–17). Interest in this has been renewed by the ability to perform detailed assessments of heterogeneous Ca^{2+} responses and network activity (9,18–20), approaches by cell sorting or single-cell molecular profiling (21–26) and approaches to connect molecular and functional heterogeneity (27–30).

In islets, we can define β-cell subpopulations that are relatively rare and with disproportionate roles in islet activity, such as "hub" and "leader" cells, and larger subpopulations defined by molecular phenotypes and sometimes functional differences in excitability, metabolism, and granularity (10,18,31). The relative abundance of these subgroups may change in type 2 diabetes (22,27,32), and across the expected human lifespan (33). Spatially, the heterogeneity of β-cell molecular markers is much less well defined. Some studies suggest localization by maturity, with the islet periphery being a site of β-cell neogenesis (34–36) and markers of maturity such as Ucn3 being more highly expressed toward the islet core (37). Given the marked differences in islet architecture, whether this is true in human islets is unclear. While mouse islets have a core-periphery structure with β-cells predominantly in the center surrounded by non-β-cell types, human islets have a more heterogeneous structure with these cell types intermixed throughout the islet, although this difference is not absolute and human-like islets can be found in mice and vice versa (38–40). Additionally, in human islets, the proportions of cell types vary considerably from islet-to-islet (38). While some authors reported a more balanced ratio of β- to non-β-cells in human islets (41), newer 3D imaging studies show that around 50% of human insulin-expressing islets might lack glucagon-producing α-cells almost completely (42,43). All these properties of human islets result in more heterotypic cell connections (44).

Despite significant and multilayered heterogeneity, β-cells operate relatively homogeneously at the tissue level. A key mechanism is electrical coupling via Connexin36 gap junction channels, which enables the propagation of intercellular waves and synchrony in response to stimulation (45–47), allowing insulin to be released in robust, coordinated pulses (4,48,49). However, due to cell-to-cell variability and the nonuniform nature of junctional coupling throughout the islet, β-cell activity manifests in complex multicellular patterns beyond what one would expect from a simple gap junctional syncytium. Intercellular waves emerge from regions of elevated excitability, change their courses, and exhibit stimulus-dependent spatiotemporal dynamics (4,18,50,51). To address the complexity of multicellular activity, research efforts often employ interdisciplinary approaches, such as integrating advanced imaging techniques with network analysis (52–54). This has led to the identification of specialized cell subpopulations, each with distinct characteristics and playing their collective role in creating complex spatiotemporal activity patterns (4,52,55). The β-cell response to stimulation is biphasic (56). In the first, so-called activation phase, first responder cells are the first to react and play a crucial role in mediating responses to increasing stimulation (19,51,57,58). Notably, during this phase, β-cells become activated in clusters whose sizes are glucose dependent, with higher glucose concentrations leading to larger coactivation domains (51). This suggests a glucose-dependent recruitment of larger functional units during activation, even before sustained oscillatory dynamics emerge. In the second, so-called sustained phase of prolonged activity, wave initiator cells (sometimes also termed as leader cells particularly when referring to drivers of slow oscillations) play a significant role by triggering intercellular excitation waves that propagate across the islet and synchronize the cells (45,50,54). As such, they presumably regulate pulsatile insulin release during this phase (18,59). In the second phase, another specialized cell group was identified—hub cells. Although our understanding of their precise functional role is still evolving, accumulating evidence suggests that they represent a subpopulation with distinct physiological and transcriptional attributes, and they may serve as central mediators of synchronized β-cell activity across the islet (4,20,54, 60,61). In contrast to wave initiator cells, their role appears to be relatively stable over time. Importantly, hub cells do not overlap with wave initiator cells (9,30,54) nor with first responder cells (51,58). Most importantly, these cell groups are not only characterized by their unique attributes and significant contributions to overall islet activity, but also by their role in shaping the nontrivial structure of functional β-cell networks. These networks are marked by heterogeneity, small-world properties, and a high degree of modularity, reflecting the presence of functional submodules (20,51,53,62). Modeling studies have shown that heterogeneity in coupling strength alone can give rise to such nontrivial network features, including small-world organization (63). It is important to note that there is also a growing interest in understanding how these subpopulations and the resulting network structures relate to cellular heterogeneity and their potential role in the development of diabetes (52,64,65).

This paper investigates the spatial organization of β-cell heterogeneity and activity within both mouse and human islets. We analyze key Ca^{2+} signaling parameters and explore how their spatial clustering correlates with the functional network structure, with particular attention to the community structure (i.e., the presence of submodules) and its potential role in shaping network behavior. Additionally, we examine the role of specific β-cell subpopulations—namely hub, wave initiator, and first responder cells—by assessing

their spatial distribution across islet communities and their influence on network activity. Through this, we aim to uncover how spatial and functional β-cell characteristics shape the collective activity within the islets of Langerhans.

MATERIALS AND METHODS

Ethics statement

In this study, we used previously published data sets derived from experiments conducted on mouse and human islets ((54) for mouse islets and (9) for human islets). While the data sets were originally generated for different research aims, this study applies novel analytical approaches to address distinct questions concerning the structure of functional β-cell networks and their relationship to spatially organized β-cell heterogeneity. Experiments on mouse islets were conducted in strict accordance with all national and European legislation (Directive 63/2010/EU) and recommendations on care and work with laboratory animals and approved by the Administration for Food Safety, Veterinary Sector and Plant Protection of the Republic of Slovenia (approval nos. U34401-12/2015/3 and U34401-35/2018-2). Experiments on human islets were performed with appropriate ethics approval from the University of Alberta Human Research Ethics Board (Pro00013094 and Pro 00001754).

Ca^{2+} imaging of islets in fresh mouse pancreatic tissue slices

Six islets of Langerhans from five male NMRI mice, aged 2–5 months were used for experiments (54). The mice were housed in ventilated cages (Allentown LLC, Allentown, NJ, USA) at temperature 22–24°C, 45–55% relative humidity, and a 12-h light/dark cycle with ad libitum access to water and standard chow (Ssniff, Soest, Germany).

After sacrificing the animal, its abdominal cavity was opened and the pancreas injected with 1.9% low-melting point agarose to support slicing on a vibratome (VT 1000 S, Leica Biosystems, Deer Park, IL). Following the extraction, 140 μm thick fresh pancreatic tissue slices were cut and loaded with Ca^{2+} dye Calbryte 520 AM (AAT Bioquest, Pleasanton, CA) using protocols and solutions as we have described in detail previously (66–69). An upright confocal microscope Leica TCS SP5 AOBS Tandem II, equipped with a 20× HCX APO L water immersion objective (NA 1.0) was used for Ca^{2+} imaging. The optical imaging thickness was approximately 5 μm at an imaging depth of two to three cell layers below the surface of the tissue slice to avoid detecting signals from superficial cells potentially damaged by the preparation procedures. Time series data were collected at 10 Hz, with resolutions of 512 × 512 pixels. The Ca^{2+}-sensitive dye was excited with a 488 nm argon laser and the Leica HyD hybrid detectors (Leica Microsystems, Wetzlar, Germany) captured the emitted fluorescence at 500–700 nm, as previously described in our studies (66,68,70). The stained slices were transferred into the recording chamber on the microscope stage, perifused continuously with carbogen-bubbled extracellular solution (ECS) at 37°C (66,68,70). After having recorded basal Ca^{2+} signals in ECS containing 6 mM glucose for 2–3 min, the ECS was exchanged with a stimulatory ECS containing 12 mM glucose (ECS-12) (8 mM glucose for supplemental figure) for 20–40 min, and returned to 6 mM ECS until the cessation of fast Ca^{2+} oscillations.

Ca^{2+} imaging in isolated human islets

Human islets were isolated in the Alberta Diabetes Institute IsletCore or the Clinical Islet Laboratory at the University of Alberta (9,71). Immediately after isolation, islets were cultured at 22°C in CMRL medium supplemented with 5 mM glucose, 100 mg/mL BSA, 0.5% insulin-transferrin-selenium (containing 10 mg/L insulin, 5.5 mg/L transferrin, and 0.0067 mg/L selenium), 100 units/mL penicillin/streptomycin, and 2 mM L-alanyl-L-glutamine dipeptide (GlutaMAX). For Ca^{2+} imaging, islets were hand-picked and cultured overnight in DMEM (Gibco) containing 5.5 mM glucose, 10% FBS, and 5% penicillin/streptomycin.

Intact islets were incubated in culture medium containing 5 mM Fluo-4 (Invitrogen) for 60 min. They were then placed in a custom recording chamber and continuously perifused with RPMI-1640 growth medium at ~32°C supplemented with 10% FBS, 5% penicillin/streptomycin, and glucose as specified. Imaging was conducted on a Zeiss SteREO Discovery V20 upright microscope equipped with a PlanApo S 3.5× mono FWD 16 mm objective and ZEN acquisition software. The excitation wavelength was 488 nm with 10% light intensity via an LED light source, and 12-bit images measuring 1388 × 1040 pixels were captured over 1 h at a rate of 0.33 Hz with a 200 ms exposure time. After having recorded the basal Ca^{2+} activity in 3 mM glucose for 10–15 min, islets were stimulated with 12 mM glucose (8 mM glucose for Fig. S2) for 30–40 min and returned to 3 mM glucose afterward. Please note that a lower baseline glucose concentration was used with isolated human islets to achieve a stable baseline without any oscillations, due to a left-shifted dose-response curve in human islets compared with mouse islets (72,73). Additionally, we wish to point out that confocal functional multicellular calcium imaging on human islets in tissue slices is not yet feasible and that, therefore, we resorted to camera-based recordings in isolated islets. The axial resolution is much less in such a system and therefore more superficial cell layers are recorded but given the distinct microarchitecture of human islets with β- and non-β-cells being present in all cell layers (see below), we believe that this methodological difference does not critically affect the obtained results.

Processing of recorded Ca^{2+} signals and analysis of β-cell activity

For the analysis of Ca^{2+} dynamics is tissue slices, regions of interest representing individual cells were manually selected using custom software called ImageFiltering (copyright Denis Špelič). Only cells with sufficiently high signal quality—allowing for individual oscillations to be clearly distinguished—and with response profiles characteristic of β-cells (i.e., quiescent at 6 mM glucose and active at 12 mM) were retained for analysis. Cells exhibiting atypical or glucose-independent activity patterns were excluded, ensuring that the analyzed population consisted predominantly of β-cells. In contrast, in isolated human islets, the discrimination of individual cells is not feasible due to limited resolution, indistinct cell borders, and the irregularity or sparsity of calcium activity in certain regions of the islet, which precludes reliable identification of individual cells based solely on morphology and signal. In this case, islet images were subdivided into a square mesh, referred to as islet subregions (ISRs), with each square having an edge length of 15 μm (9). Fluorescence intensity changes over time of each region of interest or ISR then provided the Ca^{2+} signal used for further analysis.

Response times of β-cells during stimulation were determined by manually selecting the onset of Ca^{2+} responses from the raw time series data of individual cells/ISRs. We defined the so-called first responder cells as the top 17% (1/6) of cells that reacted earliest to stimulatory glucose concentrations. To quantify sustained oscillatory activity (e.g., the sustained phase), time series from tissue slices were filtered using a zero-lag band-pass filter to extract either the fast-activity component (noise and baseline drifts) or the slow-activity component. The frequency band of interest was selected based on visual assessment, with lower and upper cutoff values set at 0.03–0.04 and 1–2 Hz, respectively. Similarly, the recordings from isolated islets underwent high-pass filtering with typical cutoff frequency of 0.005–0.01 Hz. In this way baseline drifts were eliminated. The filtered data, fast-component signals from slices and oscillatory signals from isolated islets, were additionally smoothed using adjacent averaging. Binarization was then performed by assigning a value of 1 to oscillation phases and 0 to the intervals between oscillations. Signals with extensive motion artifacts, distorted Ca^{2+} dynamics, or characteristics unrepresentative of β-cells

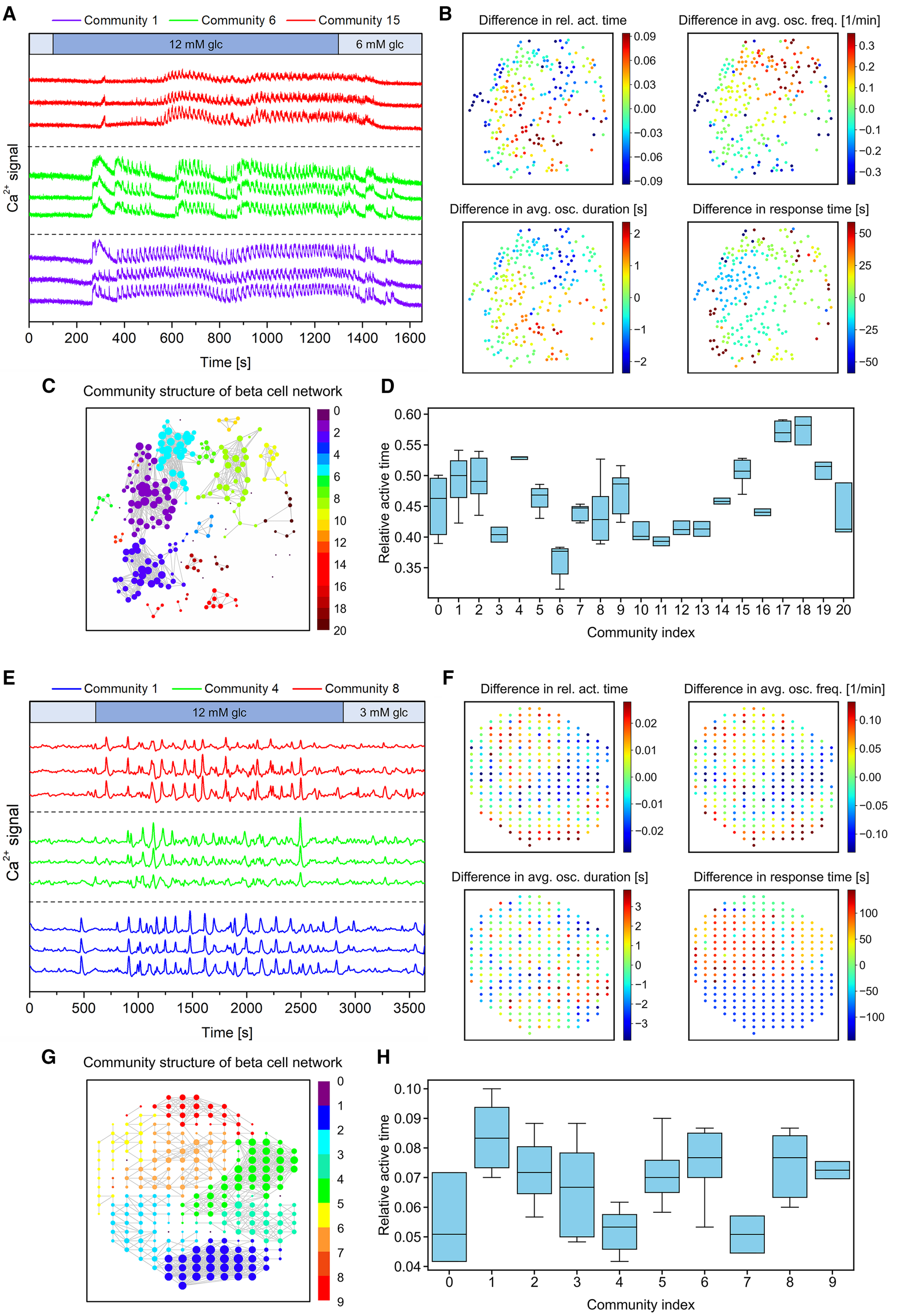

FIGURE 1 Multicellular activity and community structure of β-cell networks in a representative mouse (*A–D*) and human (*E–H*) islet. (*A* and *E*) Three Ca^{2+} signals of cells from three different communities within the representative mouse (*A*) and human (*E*) islet, as indicated by different colors (colors of signals correspond to communities in networks (*C*) and (*G*)). (*B* and *F*) β-cells within the mouse (*B*) and human (*F*) islet color-coded in accordance with the variations in different Ca^{2+} signaling parameters, expressed as the difference between the value in a given cell and the whole islet average, for the relative

(legend continued on next page)

were excluded from the analysis. The binarized signals were used to extract signaling parameters such as average oscillation frequency (f), average oscillation duration (Dur), relative active time, and to characterize intercellular Ca^{2+} waves. Specifically, the relative active time quantified the fraction of time a cell spent in the active oscillatory state. The average oscillation frequency for each cell was calculated based on the total number of oscillations within the given time interval, while the average oscillation duration was determined by averaging the lengths of all individual oscillations for that cell or ISR.

Based on binarized signals and utilizing the space-time clustering algorithm (54), we can effectively distinguish individual intercellular waves and identify the sequence of cell activations within them. From the activation sequence, we can identify cells that more frequently trigger these waves, known as wave initiator cells. Initiator cells are defined as the top 1/6 of cells that most often activated among the first within individual waves.

Functional network analysis and detection of communities

To quantify multicellular islet activity, we construct functional connectivity networks, where individual β-cells or ISR are represented as nodes, with their positions corresponding to their physical locations within the tissue (53). Connections between cells/ISRs reflect functional associations and are determined based on the temporal similarity of the measured Ca^{2+} activity. Specifically, two cells were considered functionally connected if their activity profiles exceeded a preset degree of coordinated activity, as reflected by the Pearson correlation value. We applied variable thresholds to extract the networks, aiming for an average node degree (number of connections per cell) of $k_{avg} = 8$ across all islets (74). This value is slightly higher than the electrophysiologically observed average of five to six connections, but remains within a physiologically plausible range (75) providing sufficiently dense networks for robust community detection while avoiding fragmentation in smaller islet networks. Network heterogeneity was evaluated by calculating the node degree distribution. Top 17% (1/6) of cells with the highest number of connections were identified as hub cells. Furthermore, to identify functional modules within the β-cell network, we calculated network modularity to extract communities. These communities are characterized as clusters of nodes exhibiting a higher connection density than the rest of the network. In network theory, such community structures are understood as signatures of functional segregation, reflecting the tendency of nodes to form semi-independent subgroups with stronger internal than external connectivity (76). In the context of functional connectivity networks, e.g., in neuroscience, this modular organization is typically interpreted as the presence of locally integrated units that may support parallel or partially independent processing (77). Analogously, in β-cell networks, the presence of communities suggests a structured internal organization into functional submodules with stronger intercellular similarity within than across modules. In the functional networks presented in this article, individual communities in the figures are distinguished by distinct colors.

Identifying and quantifying β-cell subpopulations

To evaluate the clustering of activity of nearby cells and of cells within individual submodules, we analyzed the distribution of relative active time within identified network communities and examined how signaling parameters vary between cell pairs based on Euclidean distance. For this purpose, we computed the absolute differences in four parameters (active time, average oscillation frequency, average oscillation duration, and response time) for all pairs of cells and compared these differences relative to their Euclidean distance. The differences were then averaged over specific distance intervals to assess how these parameters change as the distance between cells increases. The average differences for each parameter within each distance bin were calculated as follows:

$$\overline{\Delta P}(d) = \frac{1}{N(d)} \sum_{ij} \left[\Delta P_{ij} \right], \text{for } d < d_{ij} < d + \Delta d$$

where P is the analyzed parameter and $N(d)$ is the number of cell pairs with Euclidean distances in the range $[d, d + \Delta d]$.

Subsequently, we performed an analysis to quantify the differences in active response time between pairs of cells that either belong to the same community or to a different community. For each cell, we calculated the difference in signaling parameters within its local neighborhood, depending on whether the neighboring cells belonged to the same or a different community. In mouse islets, the radius was defined as 1.5 times the distance to the average distance of the six closest neighbors, while in human islets, where the distance between individual ISR is 15 μm, we defined the local neighborhood as the region within <22 μm.

To assess the overlap between detected clusters of cells with similar signaling parameters and the communities within β-cell networks, we employed hierarchical clustering. This method groups similar cells into segments, or clusters, based on distance metric. In our analysis, the distance metric was defined in three ways: Euclidean distance, differences in relative active time values, and differences in response time values between cells. Additionally, to assess the overlap between clusters from hierarchical clustering and the communities defined by Ca^{2+} signal similarity, we calculated the proportion of cells within each islet that shared both the same community index and clustering-based indices determined by the specific distance metric.

RESULTS

We employed functional multicellular Ca^{2+} imaging on mouse tissue slices and isolated human islets to record Ca^{2+} activity, as described in the materials and methods. In our experiments, we transitioned from a substimulatory (6 mM for mouse, 3 mM for human) to a stimulatory glucose concentration (12 mM). Representative Ca^{2+} signals from selected β-cells in both mouse and human islets are shown in Fig. 1, *A* and *E*. These signals reveal that, after a certain delay, the cells respond with relatively stable activity, i.e., the sustained phase. This phase of activity was used for further analyses of collective cellular dynamics. The functional β-cell networks are illustrated in Fig. 1, *C* (mouse) and *G* (human), revealing a heterogeneous and modular structure. This modular nature of the network is reflected by the presence of distinct communities (or submodules) within the network, which are highlighted by different colors that indicate to which community an individual cell

active time, average oscillation frequency, average oscillation duration and response time, as specified by the color bars. (*C* and *G*) Functional islet networks for the mouse (*C*) and the human (*G*) islet with an average network node degree $k_{avg} \approx 8$. The colors of cells indicate different communities, as specified by the color bar. (*D* and *H*) Distribution of relative active time values in different communities of the corresponding mouse (*D*) and human (*H*) islet network. Boxes of individual communities determine the 25th and 75th percentiles, whiskers denote the 10th and 90th percentiles, and the lines within boxes indicate the median values.

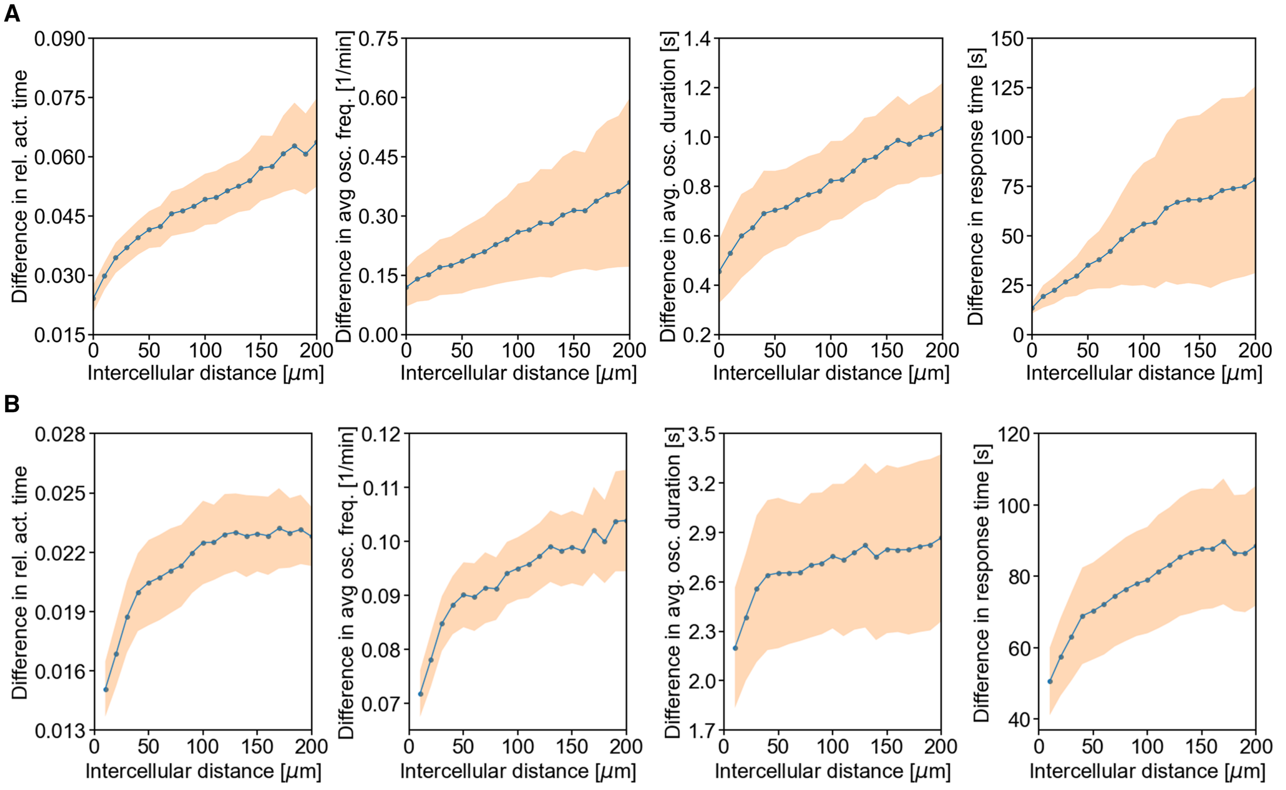

FIGURE 2 Differences in various Ca^{2+} signaling parameters as a function of intercellular distance in mouse (*A*) and human (*B*) islets. The dotted line represents the absolute difference of a given parameter between cell pairs located at a specific intercellular distance, while the shaded area indicates the corresponding standard error interval. The data are based on the average of 6 islets and 1105 cells (mouse) and 6 islets and 1344 islet subregions (human).

belongs. Corresponding Ca^{2+} traces from these communities are shown in Fig. 1, *A* and *E* and suggest a notable similarity in the signals within the same community, while differences are observed between signals from cells belonging to different communities. To evaluate intercellular differences in signaling parameters within the islets, we computed the variations in Ca^{2+} signaling metrics—such as relative active time, frequency, oscillation duration, and response time—compared with the average for the islet. Spatial distributions of these intercellular differences are displayed in Fig. 1, *B* and *F*, where in both mouse and human islets adjacent β-cells tend to display more similar signaling parameter values. Next, we further explored whether differences in signaling parameters also manifest at the level of communities within the β-cell networks. Fig. 1, *D* and *H* display the distributions of active time for all cells within a given community. We observe significant intercommunity differences in active time, with values differing by up to 60% in both the mouse and the human islet. The spatial clustering of β-cells according to their signaling characteristics prompted us to investigate this phenomenon further across a larger number of islets and to explore its relationship with the community structure of islet networks.

To assess the spatial grouping of β-cells in further detail, we calculated the average absolute differences in various Ca^{2+} signaling parameters between all cell pairs at a given intercellular distance in mouse (Fig. 2 *A*) and human (Fig. 2 *B*) islets, with data pooled from six mouse and six human islets subjected to the same protocols. We observed that differences in all signaling parameters increased with greater intercellular distance, confirming a strong tendency for cells with similar parameter values to cluster together. Notably, while this trend is observed in both mouse and human islets, a difference can be seen; in human islets, particularly when considering active time, the trend tends to saturate at distances beyond 50–100 μm, whereas in mouse islets, it continues to increase more steadily. This observation is likely related to the distinct morphology of human islets, which contain a higher proportion of heterotypic contacts with other cell types (78), making them function in a more modular manner or as islets within islets (9). Moreover, in mouse islets, the increase in overall cellular activity differences with distance is relatively larger than in human islets and, similarly, more pronounced differences are observed in response times.

In the next step, we quantified the values of relative active time and response time for β-cells within the same or

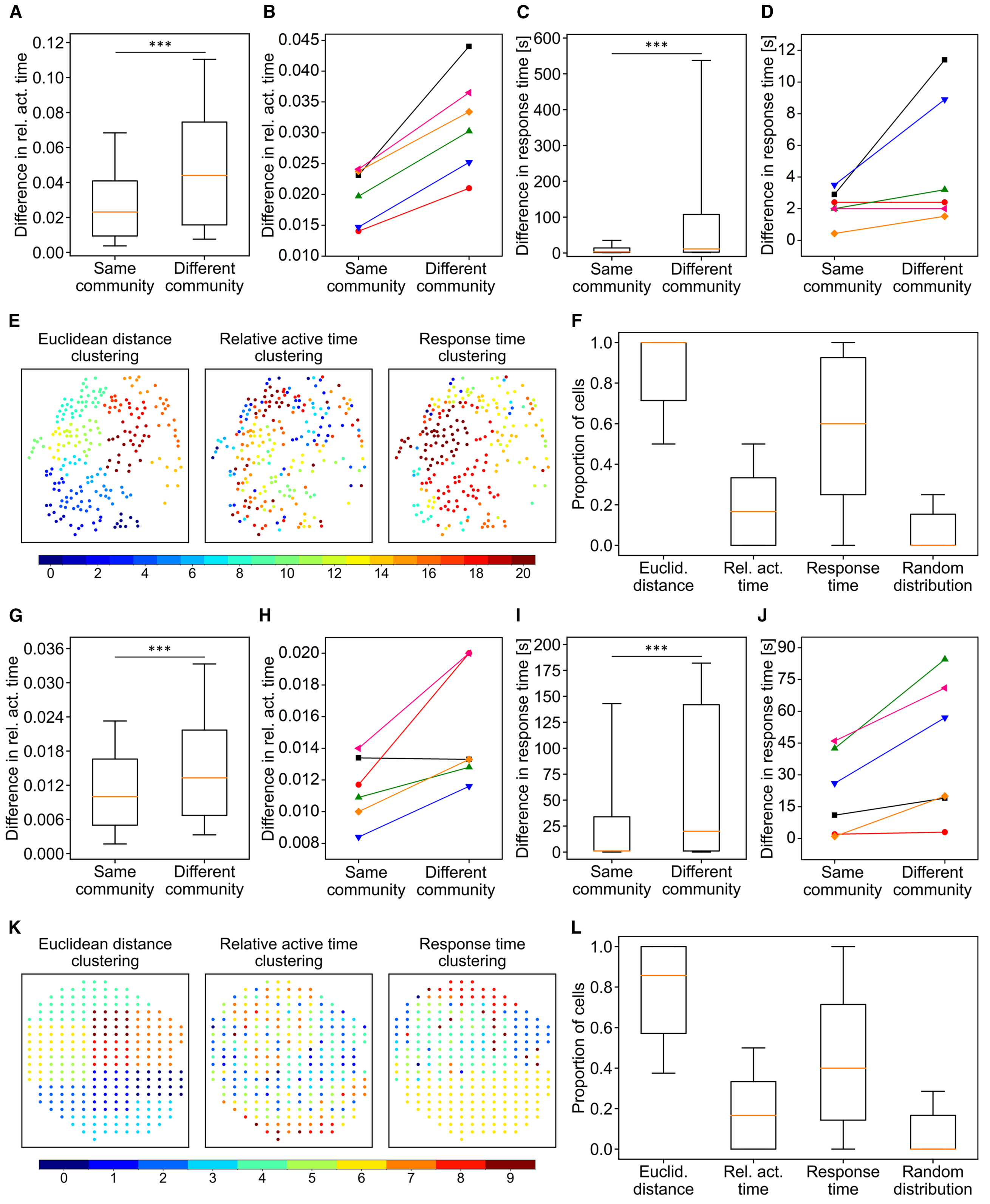

FIGURE 3 Quantification of cell clustering in mouse (*A–F*) and human (*G–L*) islets. (*A* and *G*) the distribution of differences in relative active time between all cell pairs located within the same and in different communities for a representative mouse (*A*) and human (*G*) islet. The boxes represent the interquartile range (25th to 75th percentiles), whiskers denote the 10th and 90th percentiles, and the horizontal line inside the box indicates the median value. (*B* and *H*) Differences in average relative active time values between cell pairs from the same or from different communities for six different mouse islets (*B*) and six different human islets (*H*). (*E* and *K*) Hierarchical clustering of cells in a mouse islet (*E*) and islet subregions in a human islet (*K*) based on Euclidean

(legend continued on next page)

different communities in mouse (Fig. 3, *A–D*) and human (Fig. 3, *G–J*) islets. For each cell, we calculated the average differences in active time and response time for cells located within a defined radius (i.e., 1.5 times the average of the six smallest intercellular distances) around that cell and categorized them based on whether the neighboring cells belonged to the same or different communities. Boxplots show the distribution of differences in active time (Fig. 3 *A* for mouse and Fig. 3 *G* for human) and response time (Fig. 3 *C* for mouse and Fig. 3 *I* for human) for a representative islet. Additionally, Fig. 3, *B* (mouse) and *H* (human) show the median differences in relative active time for all analyzed islets, distinguishing between proximate cell pairs that are either from the same or from a different community, while Fig. 3, *D* (mouse) and *J* (human) represent the same analysis for the response time parameter. We observed that differences in average active time are significantly smaller between pairs of cells within the same community compared with pairs from different communities, approximately 45% for mouse and 25% for human islets. A similar trend is observed in the analysis of response time differences, although values are more dispersed, especially for cells from different communities. This suggests that spatially proximate β-cells tend to share similar signaling characteristics, consistent with local functional specialization.

Next, we examined in more detail whether the clustering of cells based on activity is related to the presence of communities and which determinants shape the modular nature of β-cell networks. To this end, we used hierarchical clustering based on intercellular distances, differences in relative active time, and differences in response time values. The results of hierarchical clustering are shown in Fig. 3 *E* for mouse and Fig. 3 *K* for human islets. In both cases, the threshold determining the number of clusters was set such that the number of clusters matched the number of communities identified in Fig. 1, *C* (mouse) and *G* (human). To evaluate the overlap between clusters obtained from hierarchical clustering and the communities defined by Ca^{2+} signal similarity, we calculated the proportion of cells in a given islet with the same community index as well as the same clustering-based indices determined by Euclidean distance, relative active time values, response time values, and a random-based clustering for comparison. The results of this analysis for data pooled from six mouse and six human islets are presented in Fig. 3 *F* (mouse) and *L* (human). The results for mouse and human islets are qualitatively similar. In both cases, the highest agreement between communities and clusters is observed with clustering based on Euclidean distance, suggesting that the emergence of submodules within the islet is substantially affected by gap junctional communication between neighboring cells. A significantly higher overlap between identified clusters and network communities, compared with randomly assigned indices, is observed also for both signaling parameters—active time and response time.

Next, we sought to determine whether the segregated nature of β-cell networks influences the spatial distribution of specific cellular subpopulations, namely hub cells, first responder cells, and wave initiator cells—how these are represented within individual submodules, and whether the communities containing any of these subpopulations stand out in terms of activity. In Fig. 4, the first column shows the functional network of β-cells in a mouse islet, while the third column represents the functional network of β-cells in a human islet. Within each network, specific β-cell subgroups are highlighted in black, specifically the top 1/6 (17%) of cells with the most functional connections, i.e., hubs (Fig. 4, *A* for mouse and *C* for human), cells with the highest initiator parameter values, i.e., wave initiators (Fig. 4, *E* for mouse and *G* for human), and cells that responded first to stimulation, i.e., first responders (Fig. 4, *I* for mouse and *K* for human).

To evaluate the correlation between the presence of specific cellular subpopulations and the activity of individual communities, we present in the second and fourth columns pooled distributions of relative differences in active time for all cells from six mouse and six human islets divided into two groups—communities containing these specific cell types and communities without these specific β-cell subgroups. In mouse islets, no significant differences in activity were observed between communities that contain or do not contain the hub (Fig. 4 *B*), wave initiator (Fig. 4 *F*), and first responder (Fig. 4 *J*) cells. A slight indication of higher-than-average activity is observed in communities with wave initiators, unlike in those without, which is likely due to the above-average activity of the wave-initiating cells themselves (18,54). However, this relationship becomes blurred at the community level due to a relatively high degree of intraislet heterogeneity. In human islets, the result is quite different, as it turns out that communities containing either hubs (Fig. 4 *D*), wave initiators (Fig. 4 *H*), or first responders (Fig. 4 *L*) are significantly more active than those without. This difference, however, is less due to the above-average activity of communities with these cell subtypes and more to the below-average activity of communities that lack these subpopulations. This observation may be linked to the

distance, relative active time values, and response time values. The threshold distance for clustering was determined so that the final number of individual clusters matched the number of predefined communities in the representative islet. (*F* and *L*) The proportion of cells (*F*) and islet subregions (*L*) within a given radius (defined as 1.5 times the average of the 6 smallest intercellular distances) that share the same community index as well as the same clustering-based indices determined by Euclidean distance, relative active time values, response time values, and a random-based clustering for comparison. Data were pooled from 6 mouse islets (1105 cells) and 6 human islets (1344 islet subregions). Statistical significance: $*p < 0.05$, $**p < 0.01$, $***p < 0.001$; n.s., not significant.

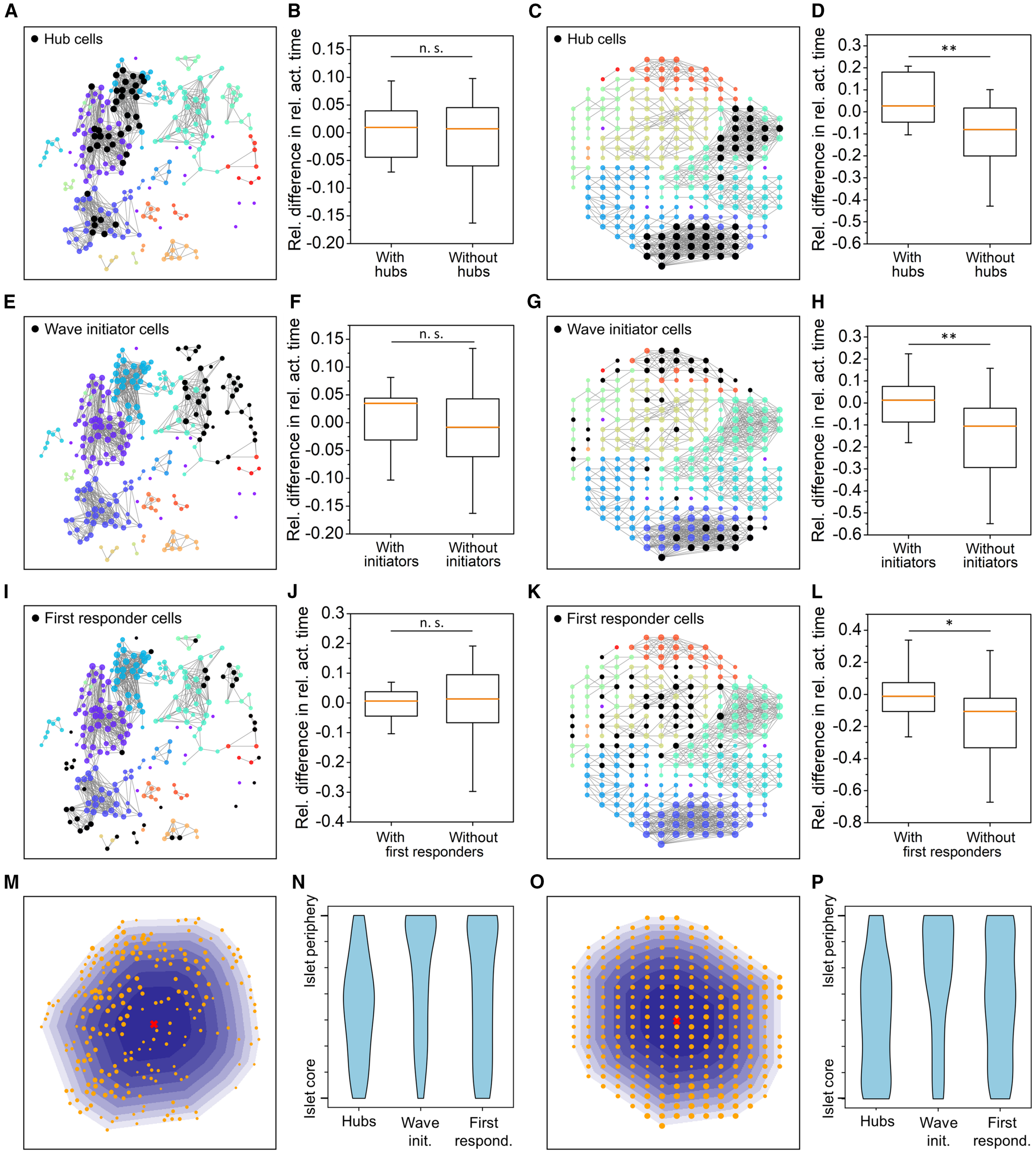

FIGURE 4 Distribution and characterization of specific β-cell subgroups: hub cells (*A–D*), wave initiator cells (*E–H*), and first responder cells (*I–L*). In the first (mouse) and third (human) column, functional islet networks are shown with a fixed average network node degree ($k_{avg} \approx 8$) with different colors indicating different communities. Black-colored cells indicate the top 1/6 of the most connected cells, i.e., hubs (mouse (*A*) and human (*C*) islets), wave initiator cells (mouse (*E*) and human (*G*) islets), and first responder cells (mouse (*I*) and human (*K*) islets). In the second (mouse) and fourth (human) column, the corresponding distributions of relative differences in active time are shown, separated by communities that contain the specific cell types (*B* and *D* for hub cells, *F* and *H* for wave indicator cells, *J* and *L* for first responder cells) and communities without these specific β-cell types. The boxes represent the 25th and 75th percentiles, whiskers denote the 10th and 90th percentiles, and the lines within the boxes indicate the median values. (*M* and *O*) Illustrate the spatial partitioning of selected mouse (*M*) and human (*O*) islets, with different color shades representing individual regions, while yellow dots indicate individual cells or subregions. (*N* mouse and *P* human) Display the distributions of specific subpopulations of cells across these regions, i.e., based on their position within the islet. The data shown represent combined results from 6 mouse islets (1105 cells) and 6 human islets (1344 islet subregions). Statistical significance: $^{*}p < 0.05$, $^{**}p < 0.01$, $^{***}p < 0.001$; n.s., not significant.

distinct morphology of human islets, where non-β-cells are often scattered within the islets.

Lastly, we analyzed the spatial distribution of hub, wave initiator, and first responder cells within islets. To achieve this, we divided mouse and human islets into eight concentric regions corresponding to the islet's shape. The outermost and largest region encompassed the entire area of the islet containing detected cells or subregions, while the remaining seven regions represented progressively smaller portions of the islet. These regions were defined so that the surface area of each was equal. This approach enabled us to quantify whether specific cell types preferentially reside in particular parts of the islet, such as central or peripheral areas. For an illustrative example of this spatial partitioning in selected mouse and human islets, see Fig. 4, *M* and *O*, respectively. We combined data from all analyzed islets to present the overall spatial distribution and depict these distributions in Fig. 4, *N* (mouse) and *P* (human). Our findings reveal that hub cells in both mouse and human islets are preferentially located in central regions and are less common in peripheral areas. Conversely, wave initiator cells are predominantly found in the peripheral regions of islets. For first responder cells, we observed a qualitative difference between mouse and human islets: in human islets, these cells are distributed homogeneously throughout the islet, whereas in mouse islets, they are preferentially located in peripheral regions closer to the mantle.

DISCUSSION

Cellular heterogeneity is fundamental to many tissues, enabling robust function and adaptability. In pancreatic islets, where β-cells must coordinate their responses to dynamic stimuli, this diversity may be critical for effective insulin secretion and metabolic control. Although considerable research has examined β-cell heterogeneity from phenotypic and functional perspectives (4,52,55), less attention has been paid to its spatial distribution. Recent studies have begun addressing the positions of critical subgroups of cells with a disproportionate influence on Ca^{2+} dynamics, such as first responders, wave initiators, and hub cells (18,30,50). However, broader, systemic principles of spatial organization and how the specialized subpopulations fit into this context remain unexplored. In our study, we address these questions and find that β-cells with similar signaling characteristics cluster spatially into larger groups. This functional clustering appears to be associated with the arrangement of intercellular coupling and, consequently, the modular structure of functional β-cell networks.

β-Cells in mouse and human islets form a syncytium through gap junctional coupling and additional paracrine mechanisms (4). While this coupling enhances collective efficiency, heterogeneity persists and may be crucial for β-cell function (9,53,79–81). In terms of functional connectivity based on network analyses, heterogeneity stems from subtle differences in Ca^{2+} signals between β-cells. It should be first pointed out that in this study as well as in the majority of previous studies by our and other groups, functional networks were constructed based on the sustained phase. In mouse islets, nearly all β-cells show synchronized fast Ca^{2+} oscillations facilitated by traveling depolarization and Ca^{2+} waves, whereas in human islets, global Ca^{2+} waves are observed only occasionally, leading to more localized synchronization (9,78,82), consistent with previous reports demonstrating that Ca^{2+} activity in human islets is often coordinated over limited spatial domains, invariant with islet size (83). These differences partly reflect distinct degrees of functional connectivity: in mouse islets, subtle variations in oscillatory timing likely emerge from spatially organized intrinsic differences, giving rise to community structure, whereas in human islets, more pronounced differences (e.g., some cells not oscillating) yield more segregated networks. Factors such as polyclonal β-cell origins, heterogeneous expression of channels and transporters, and the presence of non-β-cells creating "bottlenecks" (4,84–87) likely underlie the "islets within islets" phenomenon. Our assessment of overlap between network-based communities and clusters based on the distance between β-cells, their response, and active time revealed an excellent degree of overlap between communities and clusters based on distance, which suggests that the most important factor for Ca^{2+} signal similarity is physical proximity. In other words, whatever the most important intercellular coupling mechanism may be, i.e., gap junctional or some other type of coupling, direct neighbors show the most similar Ca^{2+} signals (88). Additionally, response time-based clusters show a considerable degree of overlap with network communities, although the first are based on the activation and the latter on the sustained phase. However, we have previously shown that during activation, β-cells are recruited in clusters of neighboring β-cells (51,68,89) and these clusters seem to correspond to a large degree to communities of β-cells with the most similar Ca^{2+} signals during the sustained phase of activity. The most straightforward explanation for this behavior may be that groups of cells belonging to such a community or cluster are more strongly coupled with each other by gap junctions than they are to other subgroups of cells, which makes them react together to stimulation and escape the clamping effect by nonactivated cells during activation, as well as enabling them to have the most similar fast Ca^{2+} oscillations with shortest intercellular delays. Videos S1 and S2 illustrate how, under 8 mM glucose, local Ca^{2+} waves remain confined within communities, whereas global waves, more common in mouse islets and sporadic in human islets, traverse multiple communities. By contrast, similarities in active time did not align as closely with network communities, reflecting instances where cells in different communities share overall activity levels but differ in oscillatory details. This may be most easily illustrated by the fact that out of a certain number

of all fast oscillations during a given time period in an islet, some communities miss out on some of them and others on some other ones (see Fig. 1).

Importantly, in human islets, where non-β-cell types such as α-cells are more interspersed within the islet interior, the observed modularity could, at least in part, be attributed to underlying anatomical compartmentalization or fewer β-to-β cell contacts. To assess this possibility, we analyzed an islet in which adrenergic stimulation was used to functionally identify regions enriched with α-cells. As shown in Fig. S1, we observed clear modular organization even outside the α-cell-rich domains, while the α-cell-dominant region formed a distinct module with relatively sparse intracommunity connectivity. These findings suggest that the modular organization in human islets is not merely an artifact of mixed cellular composition but reflects genuine functional clustering among β-cells. Furthermore, the observation that modularity is also a robust feature of mouse islets that exhibit a more uniform cytoarchitecture and a β-cell-dominant core indicates that spatial modularity is not solely driven by anatomical constraints or gap junctional coupling. Instead, it likely arises from differences in intrinsic cellular properties, which coshape the structure of functional connectivity. This implies that functional submodules reflect local similarities in signaling characteristics that persist even in the presence of relatively homogeneous spatial arrangements. Thus, cellular heterogeneity at the level of Ca^{2+} dynamics appears to be a key determinant of modularity. Nonetheless, future studies will be needed to determine whether the observed functional modularity is shaped solely by intrinsic β-cell properties or whether it may also be influenced directly or indirectly by the presence and spatial distribution of other endocrine cell types within the islet. It should also be noted that, in mouse islets, we focused solely on fast Ca^{2+} oscillations, which are well defined in tissue slices and propagate across cells in the form of intercellular waves. However, the type of oscillatory activity (i.e., fast versus slow) can significantly influence the structure of derived functional networks, as we have shown previously (74). Whether similar modularity is also a feature of the slow oscillatory component and how it may relate to spatial organization remains an open and intriguing question for future research.

In our investigation of whether and how the presence of functional submodules influences the distribution of subpopulations, i.e., hub, first responder, and wave initiator cells, we observed that, in mouse islets, their presence is not associated with the activity levels of individual submodules (Fig. 4, *B*, *F*, and *J*). The only exception was observed for wave initiators, where communities containing these cells exhibited an above-average activity (Fig. 4 *F*). This is likely because wave initiator cells themselves tend to be more active (18,54). However, due to the variability in activity across different communities and the fact that many cells in wave-initiating communities are not themselves initiators, the difference between communities with and without initiators was not statistically significant. These findings may point to a degree of functional redundancy, suggesting that the coordinated activity of the islet network does not rely exclusively on a single subpopulation or localized module, but may instead emerge from the collective dynamics of multiple communities capable of supporting similar levels of activation, a feature that could contribute to the robustness of islet function. At the same time, it is important to note that, in mouse islets, calcium activity frequently spans across modular boundaries, particularly under sustained stimulation, indicating that individual communities are not functionally autonomous. Rather, intercommunity communication likely contributes to maintaining global coherence even in the presence of spatially structured modularity. In human donor islets, however, the results were qualitatively different. All regions lacking any of the three subpopulations were found to be below average in activity and significantly less active than regions containing hub, wave initiator, or first responder cells (Fig. 4, *D*, *H*, and *L*). Although it is worth noting that, on average, none of these regions were significantly above average in activity. This difference can be attributed to the distinct cytoarchitecture in human islets, where α-, β-, and δ-cells are more heterogeneously dispersed throughout the tissue (39,72,78). Previous studies have shown that regions containing α-cells tend to exhibit lower activity and reduced functional connectivity (9). Consequently, less-active regions with non-β-cells are less likely to contain hubs or wave initiator cells, suggesting that these specialized cells are more likely to be located within neighboring communities.

Moreover, differences in the functional organization of islets, particularly the arrangement in mouse islets where β-cells are predominantly clustered in the core with α- and δ-cells forming a peripheral mantle, likely account for the observed qualitative differences in the spatial distribution of different cell subtypes. In both mouse and human islets, we identified a pronounced tendency for wave initiator cells to reside at the islet periphery (Fig. 4, *N* and *P*), a finding that aligns at least partially with previous studies (18,50,90) Furthermore, in mouse islets, first responder cells also more frequently occupy peripheral regions (Fig. 4 *N*), possibly reflecting their proximity to α-cells—a trend not observed in human islets (Fig. 4 *P*), where α-cells are more uniformly dispersed throughout the tissue. This peripheral enrichment of first responders may be explained by their reduced number of coupled β-cell neighbors due to adjacency to nonexcitable α-cells, which favors earlier activation. This interpretation aligns with the findings of Kravets et al. (58), who reported that first responders tend to exhibit lower gap junctional connectivity and higher excitability. Emerging evidence from a recent preprint shows that disrupting glucagon-GLP-1R-mediated α-β paracrine signaling delays and desynchronizes early β-cell responses, supporting a role for α-cells in promoting early

activation (91). In contrast, hub cells exhibit a different distribution pattern, occupying various positions within the islet but concentrating primarily in the central region (Fig. 4, *N* and *P*). This pattern may be consistent with their presumed role as mediators of intercellular signaling, although direct functional evidence remains limited. Their preferential central positioning also aligns with observations reported in previous studies (50). It is worth noting that our classification of these cell subtypes relied on a threshold set at the top 17% of ranked cells, which, although arbitrary, was chosen to balance statistical power and selectivity. Importantly, we have previously shown that the qualitative conclusions of our analysis remain robust when this threshold is varied (e.g., to 10 or 25%) (54).

Our study utilizes a commonly applied stimulatory glucose concentration of 12 mM, which effectively activates the majority of β-cells within the islets. However, it is important to note that the concentration of the stimulus influences β-cell responses and their collective activity. Previous studies have demonstrated that higher glucose concentrations result in shorter time lags in responses, activation occurring in larger clusters, increased activity levels during the second phase, a higher prevalence of global intercellular waves, and less modular functional networks in both mouse (51,92) and human islets (9). To explore whether the observed organizational principles and activity patterns depend on glucose concentration, we performed additional analyses using a lower 8 mM glucose stimulus. Fig. S2 presents the main findings from both mouse and human islets stimulated with 8 mM glucose, demonstrating that the results are qualitatively similar: the networks remain modular, cellular activity is spatially clustered, and significant differences in activity levels persist among individual communities.

Although spatial clustering and modularity seem robust, whether these patterns persist over time remained unclear. To address this, we split the sustained phase into two intervals, constructed networks and mapped communities for each. In both mouse (Fig. S3) and human islets (Fig. S4), the network structure, community composition, and spatial distribution of activity remained notably stable, with lower variability within communities than between them. This temporal persistence of the overall community structure implies that the functional relationships between cells, reflected in their co-oscillatory behavior, are not transient but rather represent stable organizational motifs of the islet network. Such robustness suggests that modular network architecture is a fundamental feature of β-cell coordination, reflecting both cellular heterogeneity and stable intercellular interactions. The temporal persistency of β-cell networks has in part already been a subject of interest in previous studies, particularly in relation to specific cell types, where certain subpopulations were found to exhibit higher temporal stability than others. Among these are the first responders, who represent a notably stable subpopulation. Ren et al. (93) demonstrated this in isolated mouse islets, showing that nearly identical cells consistently activated at the same times during repeated rounds of glucose stimulation. This was further supported by Delgadillo-Silva et al. (57), who observed through in vivo Ca^{2+} imaging in living zebrafish larvae that first responder cells remain stable for at least 24 h. These findings also align with results from cultured mouse islets, where Kravets et al. (58) reported substantial, although not entirely rigid, temporal stability of first responders over shorter timescales. Another highly stable subpopulation within the β-cell network is represented by hub cells. As highlighted by Rutter et al. (52) in their review article, in vivo studies involving engraftment into the anterior chamber of the mouse eye, demonstrated that hub cell populations remain stable over prolonged periods. Similar findings were reported by Šterk et al. (54), who, using acute mouse pancreatic tissue slices, found that the significant and glucose-independent temporal stability of hub cells is likely attributed to their strong gap junctional coupling. However, gap junctional coupling is not the only determinant of hub cell identity and stability. Recent studies have indicated that hub cells may also possess characteristic transcriptional and metabolic signatures associated with cellular maturity and metabolic activity (20,94). By contrast, wave initiators display lower consistency, arising stochastically in highly excitable regions (18,30,54), an observation aligned with emergent behavior models in excitable networks (95). However, the process of wave initiation depends on various factors, making the waves' sources and directions of propagation variable. Recently, Jin et al. (50) expanded these findings through 3D analyses of the entire mouse islet, demonstrating that highly synchronized hub cells remain stable both spatially and temporally, while wave initiators display a stochastic nature, with their identity rotating over time.

In this study, we demonstrate that β-cell networks are highly modular, with community structures strongly influenced by the spatial positions of cells. Adjacent β-cells with similar Ca^{2+} signaling characteristics cluster together, and their spatial organization correlates with the functional network structure. Importantly, we observe that spatial clustering is interrelated with the modular nature of the network, where distinct subpopulations such as hub, wave initiator, and first responder cells are primarily distributed across different communities. These subpopulations show context-dependent distributions, influenced by their proximity to other cell types in both mouse and human islets. This spatial organization and modularity, at least partially arising from a nonuniform distribution of intercellular coupling strengths, may serve as a foundation for robust, adaptable, and stimulus-dependent tuning of collective β-cell dynamics. Drawing a parallel with neuronal networks, modularity has been shown to enable the independent operation of subcomponents, which enhances robustness, facilitates division of labor, and supports adaptive responses to varying environmental demands (77). Similarly, in β-cell

networks, activity tends to be more localized under lower glucose concentrations or during transient stimulations (89), and the underlying functional networks exhibit a highly modular architecture (51,53). A recent study by Nasteska et al. (94) further supports the importance of cellular heterogeneity: selective elimination of immature β-cells with low PDX1 and MAFA expression led to more homogeneous islets with disrupted network architecture, absence of hub cells, and impaired insulin secretion—despite normal insulin content. These findings highlight that maintaining both transcriptional and functional heterogeneity is essential for preserving flexible and efficient islet responses to dynamic metabolic challenges (94). Similar mechanisms of spatially bound heterogeneity and functional modularity have been observed in other tissues, such as hepatocytes in liver lobules (96), neuronal populations (97), intestinal enterocytes (98), and pituitary growth hormone cells (99), where gradients of environmental factors and cell-cell interactions guide specialization and orchestrate coordinated tissue-level functions. Our findings underscore the interrelated nature of spatial clustering and functional modularity as a general organizational principle, consistently observed in both mouse and human islets. These novel insights not only enhance our understanding of β-cell networks but also provide a valuable framework for future studies aimed at unraveling the complexities of collective β-cell behavior and function in both health and disease, with potential implications for the design and composition of stem cell-derived islets.

Statistical analysis

Statistical significance of differences between groups (p values) was calculated using the Mann-Whitney U-test. Significance levels are indicated in the figures or figure legends. Asterisks denote the following significance levels: *$p < 0.05$, **$p < 0.01$, ***$p < 0.001$, and n.s., not significant.

DATA AND CODE AVAILABILITY

The source code and tools for β-cell signal processing and network analysis are available at https://github.com/MarkoSterk/beta_cell_analysis_suite.

ACKNOWLEDGMENTS

The work presented in this study was financially supported by the Slovenian Research and Innovation Agency (research core funding nos. P3-0396 and I0-0029, as well as research projects nos. J3- 60062, L3-60156, J3-3077). Work on human islets was funded in part by a Human Islet Research Network (HIRN) grant from the National Institutes of Health (U01DK120447). P.E.M. holds the Canada Research Chair in Islet Biology.

AUTHOR CONTRIBUTIONS

Conceptualization, M.D., P.E.M., A.S., and M.G.; methodology, M.D. and L.K.B.; investigation, M.D. and M.G.; formal analysis, M.D.; visualization, M.D.; writing – original draft, M.D., P.E.M., A.S., and M.G.; writing – review & editing, M.D., L.K.B., P.E.M., and M.G.; software, M.Š.; data curation, M.Š.; supervision, M.G.

DECLARATION OF INTERESTS

The authors declare no conflict of interest.

SUPPORTING MATERIAL

Supporting material can be found online at https://doi.org/10.1016/j.bpj.2025.07.043.

REFERENCES

1. Hasenauer, J., C. Hasenauer, ..., F. J. Theis. 2014. ODE Constrained Mixture Modelling: A Method for Unraveling Subpopulation Structures and Dynamics. *PLoS Comput. Biol.* 10:e1003686.
2. Kim, C., G. J. Seedorf, ..., D. P. Shepherd. 2019. Heterogeneous response of endothelial cells to insulin like growth factor 1 treatment is explained by spatially clustered subpopulations. *Biol. Open.* 8:bio045906.
3. Carter, B., and K. Zhao. 2021. The epigenetic basis of cellular heterogeneity. *Nat. Rev. Genet.* 22:235–250.
4. Benninger, R. K. P., and V. Kravets. 2022. The physiological role of β-cell heterogeneity in pancreatic islet function. *Nat. Rev. Endocrinol.* 18:9–22.
5. Ferdaoussi, M. 2024. Metabolic and Molecular Amplification of Insulin Secretion. *Adv. Anat. Embryol. Cell Biol.* 239:117–139.
6. Tokarz, V. L., P. E. MacDonald, and A. Klip. 2018. The cell biology of systemic insulin function. *J. Cell Biol.* 217:2273–2289.
7. Merrins, M. J., B. E. Corkey, ..., M. Prentki. 2022. Metabolic cycles and signals for insulin secretion. *Cell Metab.* 34:947–968.
8. Marinelli, I., P. A. Fletcher, ..., R. Bertram. 2021. Symbiosis of Electrical and Metabolic Oscillations in Pancreatic β-Cells. *Front. Physiol.* 12:781581.
9. Gosak, M., R. Yan-Do, ..., A. Stožer. 2022. Ca2+ Oscillations, Waves, and Networks in Islets From Human Donors With and Without Type 2 Diabetes. *Diabetes.* 71:2584–2596.
10. Van Schravendijk, C. F., R. Kiekens, and D. G. Pipeleers. 1992. Pancreatic beta cell heterogeneity in glucose-induced insulin secretion. *J. Biol. Chem.* 267:21344–21348.
11. Pipeleers, D. G. 1992. Heterogeneity in pancreatic beta-cell population. *Diabetes.* 41:777–781.
12. Salomon, D., and P. Meda. 1986. Heterogeneity and contact-dependent regulation of hormone secretion by individual B cells. *Exp. Cell Res.* 162:507–520.
13. Stefan, Y., P. Meda, ..., L. Orci. 1987. Stimulation of insulin secretion reveals heterogeneity of pancreatic B cells in vivo. *J. Clin. Investig.* 80:175–183.
14. Bertram, R., J. Previte, ..., L. S. Satin. 2000. The phantom burster model for pancreatic beta-cells. *Biophys. J.* 79:2880–2892.
15. Heimberg, H., A. De Vos, ..., F. Schuit. 1993. Heterogeneity in glucose sensitivity among pancreatic beta-cells is correlated to differences in glucose phosphorylation rather than glucose transport. *EMBO J.* 12:2873–2879.
16. Zhang, M., P. Goforth, ..., L. Satin. 2003. The Ca2+ dynamics of isolated mouse beta-cells and islets: implications for mathematical models. *Biophys. J.* 84:2852–2870.
17. Aldous, N., A. S. M. Moin, and E. M. Abdelalim. 2023. Pancreatic β-cell heterogeneity in adult human islets and stem cell-derived islets. *Cell. Mol. Life Sci.* 80:176.

18. Westacott, M. J., N. W. F. Ludin, and R. K. P. Benninger. 2017. Spatially Organized β-Cell Subpopulations Control Electrical Dynamics across Islets of Langerhans. *Biophys. J.* 113:1093–1108.

19. Salem, V., L. D. Silva, …, G. A. Rutter. 2019. Leader β-cells coordinate Ca^{2+} dynamics across pancreatic islets in vivo. *Nat. Metab.* 1:615–629.

20. Johnston, N. R., R. K. Mitchell, …, D. J. Hodson. 2016. Beta Cell Hubs Dictate Pancreatic Islet Responses to Glucose. *Cell Metab.* 24:389–401.

21. Segerstolpe, Å., A. Palasantza, …, R. Sandberg. 2016. Single-Cell Transcriptome Profiling of Human Pancreatic Islets in Health and Type 2 Diabetes. *Cell Metab.* 24:593–607.

22. Dorrell, C., J. Schug, …, M. Grompe. 2016. Human islets contain four distinct subtypes of β cells. *Nat. Commun.* 7:11756.

23. Enge, M., H. E. Arda, …, S. R. Quake. 2017. Single-Cell Analysis of Human Pancreas Reveals Transcriptional Signatures of Aging and Somatic Mutation Patterns. *Cell.* 171:321–330.e14.

24. Li, J., J. Klughammer, …, S. Kubicek. 2016. Single-cell transcriptomes reveal characteristic features of human pancreatic islet cell types. *EMBO Rep.* 17:178–187.

25. Chiou, J., R. J. Geusz, …, K. J. Gaulton. 2021. Interpreting type 1 diabetes risk with genetics and single-cell epigenomics. *Nature.* 594:398–402.

26. Chiou, J., C. Zeng, …, K. J. Gaulton. 2021. Single-cell chromatin accessibility identifies pancreatic islet cell type- and state-specific regulatory programs of diabetes risk. *Nat. Genet.* 53:455–466.

27. Wang, G., J. Chiou, …, M. Sander. 2023. Integrating genetics with single-cell multiomic measurements across disease states identifies mechanisms of beta cell dysfunction in type 2 diabetes. *Nat. Genet.* 55:984–994.

28. Dai, X.-Q., J. Camunas-Soler, …, P. E. MacDonald. 2022. Heterogenous impairment of α cell function in type 2 diabetes is linked to cell maturation state. *Cell Metab.* 34:256–268.e5.

29. Camunas-Soler, J., X.-Q. Dai, …, P. E. MacDonald. 2020. Patch-Seq Links Single-Cell Transcriptomes to Human Islet Dysfunction in Diabetes. *Cell Metab.* 31:1017–1031.e4.

30. Chabosseau, P., F. Yong, …, G. A. Rutter. 2023. Molecular phenotyping of single pancreatic islet leader beta cells by "Flash-Seq". *Life Sci.* 316:121436.

31. Rui, J., S. Deng, …, K. C. Herold. 2017. β Cells that Resist Immunological Attack Develop during Progression of Autoimmune Diabetes in NOD Mice. *Cell Metab.* 25:727–738.

32. Wang, Y. J., M. L. Golson, …, K. H. Kaestner. 2016. Single-Cell Mass Cytometry Analysis of the Human Endocrine Pancreas. *Cell Metab.* 24:616–626.

33. Shrestha, S., G. Erikson, …, R. Arrojo E Drigo. 2022. Aging compromises human islet beta cell function and identity by decreasing transcription factor activity and inducing ER stress. *Sci. Adv.* 8:eabo3932.

34. Lee, S., J. Zhang, …, M. O. Huising. 2021. Virgin β-Cells at the Neogenic Niche Proliferate Normally and Mature Slowly. *Diabetes.* 70:1070–1083.

35. van der Meulen, T., A. M. Mawla, …, M. O. Huising. 2017. Virgin Beta Cells Persist throughout Life at a Neogenic Niche within Pancreatic Islets. *Cell Metab.* 25:911–926.e6.

36. Huising, M. O., S. Lee, and T. van der Meulen. 2018. Evidence for a Neogenic Niche at the Periphery of Pancreatic Islets. *Bioessays.* 40:e1800119.

37. Li, F., D. Hu, …, G. Orr. 2021. Single Molecule-Based fliFISH Validates Radial and Heterogeneous Gene Expression Patterns in Pancreatic Islet β-Cells. *Diabetes.* 70:1117–1122.

38. Brissova, M., M. J. Fowler, …, A. C. Powers. 2005. Assessment of Human Pancreatic Islet Architecture and Composition by Laser Scanning Confocal Microscopy. *J. Histochem. Cytochem.* 53:1087–1097.

39. Hoang, D. T., H. Matsunari, …, J. Jo. 2014. A Conserved Rule for Pancreatic Islet Organization. *PLoS One.* 9:e110384.

40. Dolenšek, J., M. S. Rupnik, and A. Stožer. 2015. Structural similarities and differences between the human and the mouse pancreas. *Islets.* 7:e1024405.

41. Kim, A., K. Miller, …, M. Hara. 2009. Islet architecture: A comparative study. *Islets.* 1:129–136.

42. Lehrstrand, J., W. I. L. Davies, …, U. Ahlgren. 2024. Illuminating the complete ß-cell mass of the human pancreas- signifying a new view on the islets of Langerhans. *Nat. Commun.* 15:3318.

43. Hahn, M., C. Nord, …, U. Ahlgren. 2021. 3D imaging of human organs with micrometer resolution - applied to the endocrine pancreas. *Commun. Biol.* 4:1063.

44. Félix-Martínez, G. J., and J. R. Godínez-Fernández. 2022. Comparative analysis of reconstructed architectures from mice and human islets. *Islets.* 14:23–35.

45. Benninger, R. K. P., M. Zhang, …, D. W. Piston. 2008. Gap Junction Coupling and Calcium Waves in the Pancreatic Islet. *Biophys. J.* 95:5048–5061.

46. Meda, P. 2012. The In Vivo β-to-β-Cell Chat Room: Connexin Connections Matter. *Diabetes.* 61:1656–1658.

47. Ng, X. W., Y. H. Chung, and D. W. Piston. 2021. Intercellular Communication in the Islet of Langerhans in Health and Disease. *Compr. Physiol.* 11:2191–2225.

48. Bosco, D., J. A. Haefliger, and P. Meda. 2011. Connexins: Key mediators of endocrine function. *Physiol. Rev.* 91:1393–1445.

49. Benninger, R. K. P., and D. W. Piston. 2014. Cellular communication and heterogeneity in pancreatic islet insulin secretion dynamics. *Trends Endocrinol. Metab.* 25:399–406.

50. Jin, E., J. K. Briggs, …, M. J. Merrins. 2025. Glucokinase activity controls peripherally located subpopulations of β-cells that lead islet Ca2+ oscillations. *eLife.* 13:RP103068.

51. Stožer, A., M. Skelin Klemen, …, J. Dolenšek. 2021. Glucose-dependent activation, activity, and deactivation of beta cell networks in acute mouse pancreas tissue slices. *Am. J. Physiol. Endocrinol. Metab.* 321:E305–E323.

52. Rutter, G. A., A. Gresch, …, R. K. P. Benninger. 2024. Exploring pancreatic beta-cell subgroups and their connectivity. *Nat. Metab.* 6:2039–2053.

53. Stožer, A., M. Šterk, …, M. Gosak. 2022. From Isles of Königsberg to Islets of Langerhans: Examining the Function of the Endocrine Pancreas Through Network Science. *Front. Endocrinol.* 13:922640.

54. Šterk, M., J. Dolenšek, …, M. Gosak. 2023. Functional characteristics of hub and wave-initiator cells in β cell networks. *Biophys. J.* 122:784–801.

55. Peercy, B. E., and A. S. Sherman. 2022. Do oscillations in pancreatic islets require pacemaker cells? *J. Biosci.* 47:14.

56. Jaffredo, M., E. Bertin, …, M. Raoux. 2021. Dynamic Uni- and Multicellular Patterns Encode Biphasic Activity in Pancreatic Islets. *Diabetes.* 70:878–888.

57. Delgadillo-Silva, L. F., E. Tasöz, …, N. Ninov. 2024. Optogenetic β cell interrogation in vivo reveals a functional hierarchy directing the Ca2+ response to glucose supported by vitamin B6. *Sci. Adv.* 10:eado4513.

58. Kravets, V., J. M. Dwulet, …, R. K. P. Benninger. 2022. Functional architecture of pancreatic islets identifies a population of first responder cells that drive the first-phase calcium response. *PLoS Biol.* 20:e3001761.

59. Benninger, R. K. P., T. Hutchens, …, D. W. Piston. 2014. Intrinsic Islet Heterogeneity and Gap Junction Coupling Determine Spatiotemporal Ca2+ Wave Dynamics. *Biophys. J.* 107:2723–2733.

60. Loppini, A., and L. Chiodo. 2019. Biophysical modeling of β-cells networks: Realistic architectures and heterogeneity effects. *Biophys. Chem.* 254:106247.

61. Briggs, J. K., A. Gresch, …, R. K. P. Benninger. 2023. β-cell intrinsic dynamics rather than gap junction structure dictates subpopulations in the islet functional network. *eLife.* 12:e83147.

62. Markovič, R., A. Stožer, …, M. S. Rupnik. 2015. Progressive glucose stimulation of islet beta cells reveals a transition from segregated to integrated modular functional connectivity patterns. *Sci. Rep.* 5:7845.

63. Cappon, G., and M. G. Pedersen. 2016. Heterogeneity and nearest-neighbor coupling can explain small-worldness and wave properties in pancreatic islets. *Chaos.* 26:053103.

64. Zhang, S., Z. Qi, …, D. Zhu. 2023. Effect of sodium-glucose transporter 2 inhibitors on sarcopenia in patients with type 2 diabetes mellitus: A systematic review and meta-analysis. *Front. Endocrinol.* 14:1203666.

65. Suba, K., Y. Patel, …, V. Salem. 2024. Intra-islet glucagon signalling regulates beta-cell connectivity, first-phase insulin secretion and glucose homoeostasis. *Mol. Metab.* 85:101947.

66. Stožer, A., J. Dolenšek, …, M. S. Klemen. 2021. Confocal Laser Scanning Microscopy of Calcium Dynamics in Acute Mouse Pancreatic Tissue Slices. *JoVE J.* 170:e62293.

67. Speier, S., and M. Rupnik. 2003. A novel approach to in situ characterization of pancreatic β-cells. *Pflugers Arch.* 446:553–558.

68. Stožer, A., J. Dolenšek, and M. S. Rupnik. 2013. Glucose-Stimulated Calcium Dynamics in Islets of Langerhans in Acute Mouse Pancreas Tissue Slices. *PLoS One.* 8:e54638.

69. Stožer, A., M. Gosak, …, D. Korošak. 2013. Functional Connectivity in Islets of Langerhans from Mouse Pancreas Tissue Slices. *PLoS Comput. Biol.* 9:e1002923.

70. Stožer, A., J. Dolenšek, …, M. Skelin Klemen. 2021. Calcium imaging. *In* Imaging Modalities for Biological and Preclinical Research: A Compendium, Volume 1: Part I: Ex Vivo Biological Imaging, A. Walter, J.G. Mannheim, and C.J. Caruana, eds. (IOP Publishing), pp. I.1.e-1–I.1.e-13.

71. Mummey, H. M., W. Elison, …, K. J. Gaulton. 2024. Single cell multiome profiling of pancreatic islets reveals physiological changes in cell type-specific regulation associated with diabetes risk. Preprint at bioRxiv. https://doi.org/10.1101/2024.08.03.606460.

72. Skelin Klemen, M., J. Dolenšek, …, A. Stožer. 2017. The triggering pathway to insulin secretion: Functional similarities and differences between the human and the mouse β cells and their translational relevance. *Islets.* 9:109–139.

73. Rodriguez-Diaz, R., R. D. Molano, …, P. O. Berggren. 2018. Paracrine Interactions within the Pancreatic Islet Determine the Glycemic Set Point. *Cell Metab.* 27:549–558.e4.

74. Šterk, M., Y. Zhang, …, M. Gosak. 2024. Network representation of multicellular activity in pancreatic islets: Technical considerations for functional connectivity analysis. *PLoS Comput. Biol.* 20:e1012130.

75. Zhang, Q., J. Galvanovskis, …, P. Rorsman. 2008. Cell coupling in mouse pancreatic β-cells measured in intact islets of Langerhans. *Philos. Trans. A Math. Phys. Eng. Sci.* 366:3503–3523.

76. Girvan, M., and M. E. J. Newman. 2002. Community structure in social and biological networks. *Proc. Natl. Acad. Sci. USA.* 99:7821–7826.

77. Sporns, O., and R. F. Betzel. 2016. Modular brain networks. *Annu. Rev. Psychol.* 67:613–640.

78. Cabrera, O., D. M. Berman, …, A. Caicedo. 2006. The unique cytoarchitecture of human pancreatic islets has implications for islet cell function. *Proc. Natl. Acad. Sci. USA.* 103:2334–2339.

79. Benninger, R. K. P., and D. J. Hodson. 2018. New Understanding of β-Cell Heterogeneity and In Situ Islet Function. *Diabetes.* 67:537–547.

80. Roscioni, S. S., A. Migliorini, …, H. Lickert. 2016. Impact of islet architecture on β-cell heterogeneity, plasticity and function. *Nat. Rev. Endocrinol.* 12:695–709.

81. Nasteska, D., and D. J. Hodson. 2018. The role of beta cell heterogeneity in islet function and insulin release. *J. Mol. Endocrinol.* 61:R43–R60.

82. Quesada, I., M. G. Todorova, …, B. Soria. 2006. Glucose induces opposite intracellular Ca2+ concentration oscillatory patterns in identified {alpha}- and β-cells within intact human islets of Langerhans. *Diabetes.* 55:2463–2469.

83. Westacott, M. J., N. L. Farnsworth, …, R. K. P. Benninger. 2017. Age-dependent decline in the coordinated [Ca^{2+}] and insulin secretory dynamics in human pancreatic islets. *Diabetes.* 66:2436–2445.

84. Scharfmann, R., X. Xiao, …, P. Ravassard. 2008. Beta Cells within Single Human Islets Originate from Multiple Progenitors. *PLoS One.* 3:e3559.

85. Farnsworth, N. L., A. Hemmati, …, R. K. P. Benninger. 2014. Fluorescence recovery after photobleaching reveals regulation and distribution of connexin36 gap junction coupling within mouse islets of Langerhans. *J. Physiol.* 592:4431–4446.

86. Slak Rupnik, M., and M. Hara. 2024. Local Dialogues Between the Endocrine and Exocrine Cells in the Pancreas. *Diabetes.* 73:533–541.

87. Bosco, D., M. Armanet, …, T. Berney. 2010. Unique Arrangement of α- and β-Cells in Human Islets of Langerhans. *Diabetes.* 59:1202–1210.

88. Dolenšek, J., V. Pohorec, …, A. Stožer. 2025. Ultrafast multicellular calcium imaging of calcium spikes in mouse beta cells in tissue slices. *Acta Physiol.* 241:e14261.

89. Gosak, M., A. Stožer, …, M. Marhl. 2017. Critical and Supercritical Spatiotemporal Calcium Dynamics in Beta Cells. *Front. Physiol.* 8:1106.

90. Dwulet, J. M., N. W. F. Ludin, …, R. K. P. Benninger. 2019. How Heterogeneity in Glucokinase and Gap-Junction Coupling Determines the Islet [Ca^{2+}] Response. *Biophys. J.* 117:2188–2203.

91. Balasenthilkumaran, N. V., L. K. Bombek, …, V. Kravets. 2025. Role of GLP1-receptor-mediated α-β-cell communication in functional β-cell heterogeneity. Preprint at bioRxiv. https://doi.org/10.1101/2025.04.25.650736.

92. Stožer, A., R. Markovič, …, M. Gosak. 2019. Heterogeneity and delayed activation as hallmarks of self-organization and criticality in excitable tissue. *Front. Physiol.* 10:869.

93. Ren, H., Y. Li, …, C. Tang. 2022. Pancreatic α and β cells are globally phase-locked. *Nat. Commun.* 13:3721.

94. Nasteska, D., N. H. F. Fine, …, D. J. Hodson. 2021. PDX1LOW MAFALOW β-cells contribute to islet function and insulin release. *Nat. Commun.* 12:674.

95. Scialla, S., A. Loppini, …, E. Heinsalu. 2021. Hubs, diversity, and synchronization in FitzHugh-Nagumo oscillator networks: Resonance effects and biophysical implications. *Phys. Rev. E.* 103:052211.

96. Ben-Moshe, S., and S. Itzkovitz. 2019. Spatial heterogeneity in the mammalian liver. *Nat. Rev. Gastroenterol. Hepatol.* 16:395–410.

97. Feldt Muldoon, S., I. Soltesz, and R. Cossart. 2013. Spatially clustered neuronal assemblies comprise the microstructure of synchrony in chronically epileptic networks. *Proc. Natl. Acad. Sci. USA.* 110:3567–3572.

98. Adler, M., N. Moriel, …, M. Nitzan. 2023. Emergence of division of labor in tissues through cell interactions and spatial cues. *Cell Rep.* 42:112412.

99. Sanchez-Cardenas, C., P. Fontanaud, …, P. Mollard. 2010. Pituitary growth hormone network responses are sexually dimorphic and regulated by gonadal steroids in adulthood. *Proc. Natl. Acad. Sci. USA.* 107:21878–21883.

Biophysical Journal, Volume *124*

Supplemental information

Spatially bound functional heterogeneity drives modular organization in β-cell networks

Maja Duh, Marko Šterk, Lidija Križančić Bombek, Patrick E. MacDonald, Andraž Stožer, and Marko Gosak

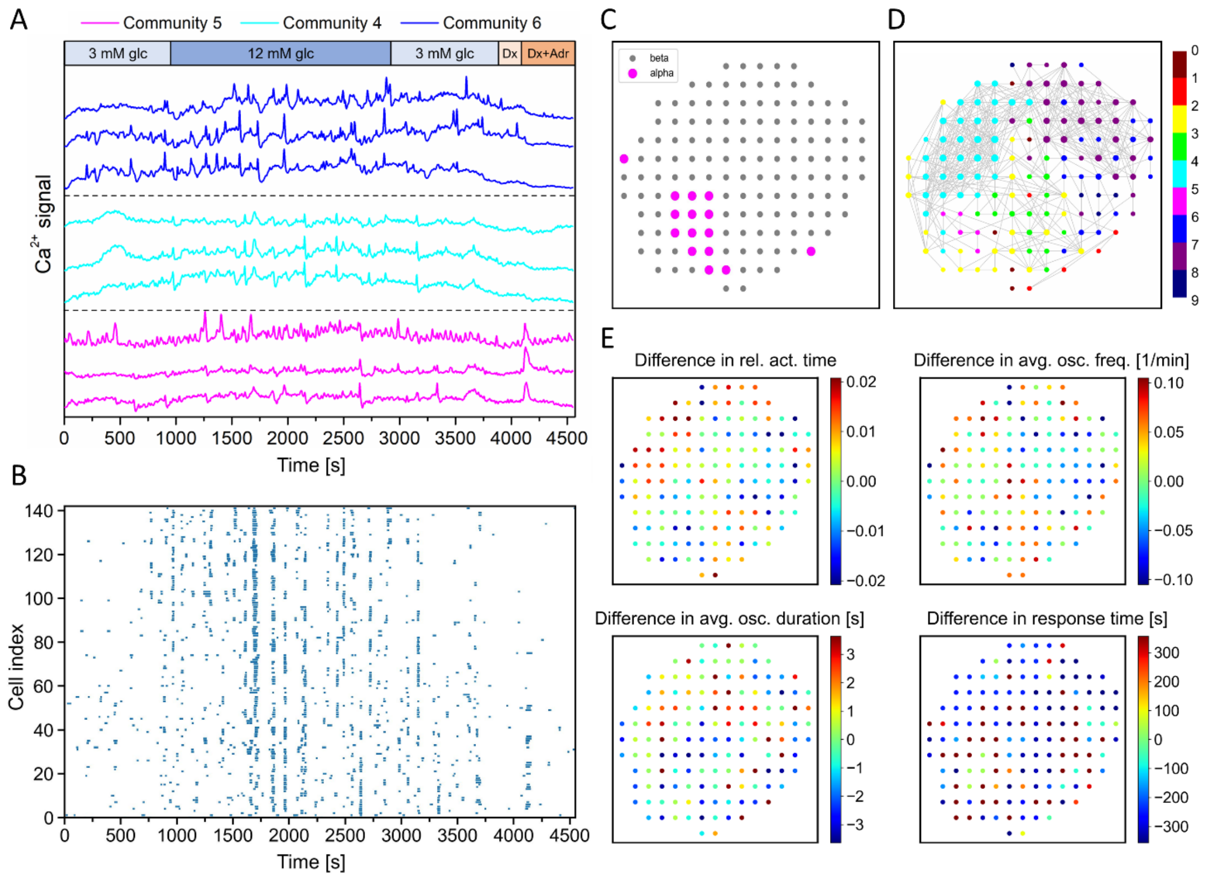

Figure S1. Multicellular activity and community structure of alpha–beta cell networks in a representative human islet. (A) Representative Ca^{2+} traces from cells belonging to three different communities within the human islet, indicated by distinct colors (trace colors correspond to the community colors in the network shown in panel D). The first three traces (magenta, Community 5) originate from a community composed of alpha cells, while the remaining traces (cyan and blue, Communities 4 and 6) correspond to beta-cell communities. Cells were first incubated in 3 mM glucose, followed by stimulation with 12 mM glucose for 30 minutes, and then returned to 3 mM glucose. After 15 minutes, the islet was briefly exposed to 3 mM glucose supplemented with 100 µM Diazoxide, followed by stimulation with 3 mM glucose + 100 µM Diazoxide + 10 µM Adrenaline. (B) Raster plot showing binarized Ca^{2+} activity of all recorded cells across the stimulation protocol. (C) Spatial distribution of alpha (magenta) and beta (gray) cells within the representative human islet. (D) Functional network of the human islet, constructed using an average node degree of $k_{avg} \approx 8$. Node colors represent distinct communities as defined by modularity analysis (see color bar). (E) Alpha and beta cells color-coded according to deviations from the islet-wide average for different Ca^{2+} signaling parameters: relative active time, average oscillation frequency, average oscillation duration, and response time (as indicated by the respective color scales).

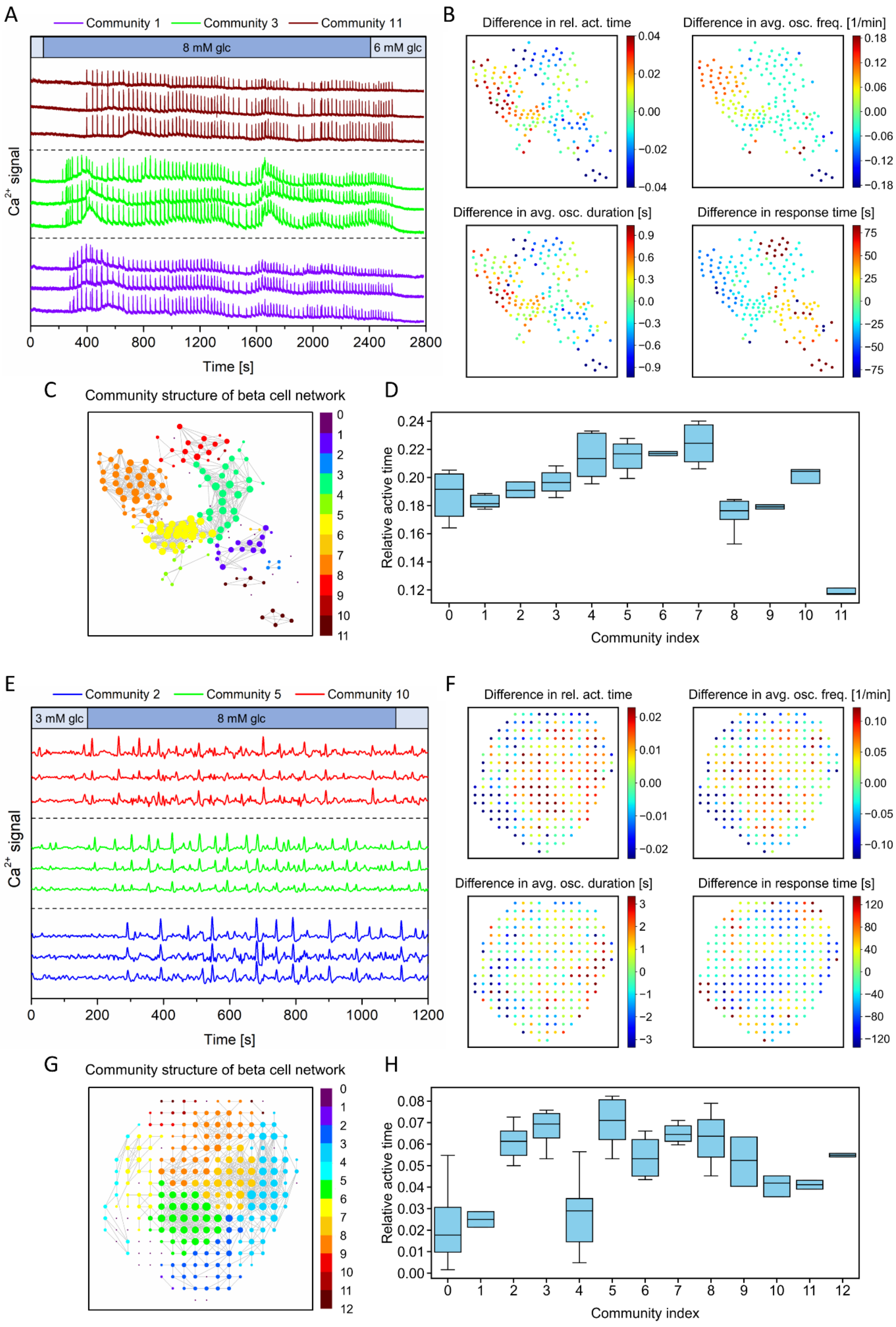
A
Community 1
Community 3
Community 11
8 mM glc
6 mM glc
Ca2+ signal
Time [s]
B
Difference in rel. act. time
Difference in avg. osc. freq. [1/min]
Difference in avg. osc. duration [s]
Difference in response time [s]
C
Community structure of beta cell network
D
Relative active time
Community index
E
Community 2
Community 5
Community 10
3 mM glc
8 mM glc
Ca2+ signal
Time [s]
F
Difference in rel. act. time
Difference in avg. osc. freq. [1/min]
Difference in avg. osc. duration [s]
Difference in response time [s]
G
Community structure of beta cell network
H
Relative active time
Community index

Figure S2: Multicellular activity and community structure of beta cell networks in representative mouse (panels A-D) and human (panels E-H) islets. (A) and (E) Three Ca^{2+} signals of cells from three different communities within the representative mouse (A) and human (E) islet, as indicated by different colors (colors on signals correspond to communities in networks (C) and (G)). (B) and (F) Beta cells within mouse (B) and human (F) islet color-coded in accordance with the variations in different Ca^{2+} signalling parameters, expressed as the difference of the value in a given cell from the whole islet average, for: the relative active time, average oscillation frequency, average oscillation duration and response time, as specified by the color bars. (C) and (G) Functional islet networks for mouse (C) and human (G) islet with an average network node degree ($k_{avg} \approx 8$). Colors of cells indicate different communities, as specified by the color bar. (D) and (H) Distribution of relative active time values in different communities of the corresponding mouse (D) and human (H) islet network. Boxes of individual communities determine the 25th and 75th percentile, whiskers denote the 10th and 90th percentile, and the lines within boxes indicate the median values.

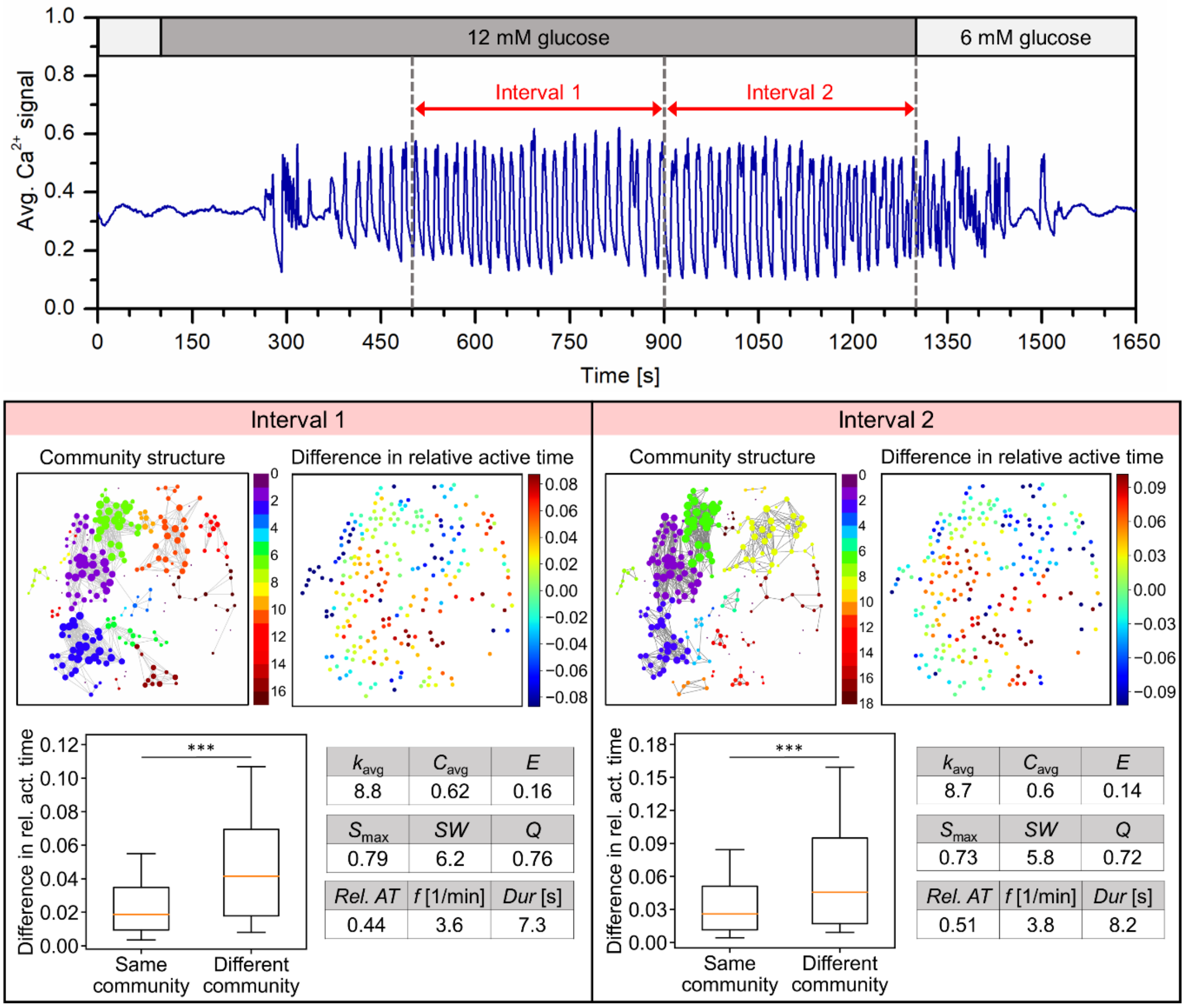

Figure S3: Temporal persistency of spatial distribution of functional heterogeneity in beta cell networks in a mouse islet. The upper panel shows the average Ca^{2+} signal from all cells within the islet, stimulated with 12 mM glucose, with designated time intervals used for the analysis. The lower panel is divided into Interval 1 and Interval 2, which were used for further analysis. For each time interval, functional beta cell networks were constructed. In these networks, colored dots represent the physical locations of cells within the islet, grey lines indicate functional connections, and the colors of the cells denote different communities. Next to this, cells within the islet are color-coded according to their differences in relative active time, as specified by the color bar. Individual values are defined as the difference between the active time of a single cell and the average active time of the entire islet. Additionally, the differences in active time within the same and different communities are shown for both intervals. Tables on the right summarize the calculated network parameters: average node degree (k_{avg}), average clustering coefficient (C_{avg}), global efficiency (*E)*, relative size of the largest component (S_{max}), small-world coefficient (*SW*), and modularity (*Q*). It also includes key cell activity parameters: relative active time (*AT*), average oscillation frequency (*f*), and average oscillation duration (*Dur*). Statistical significance: ∗$p < 0.05$, ∗∗$p < 0.01$, ∗∗∗$p < 0.001$; n.s., not significant.

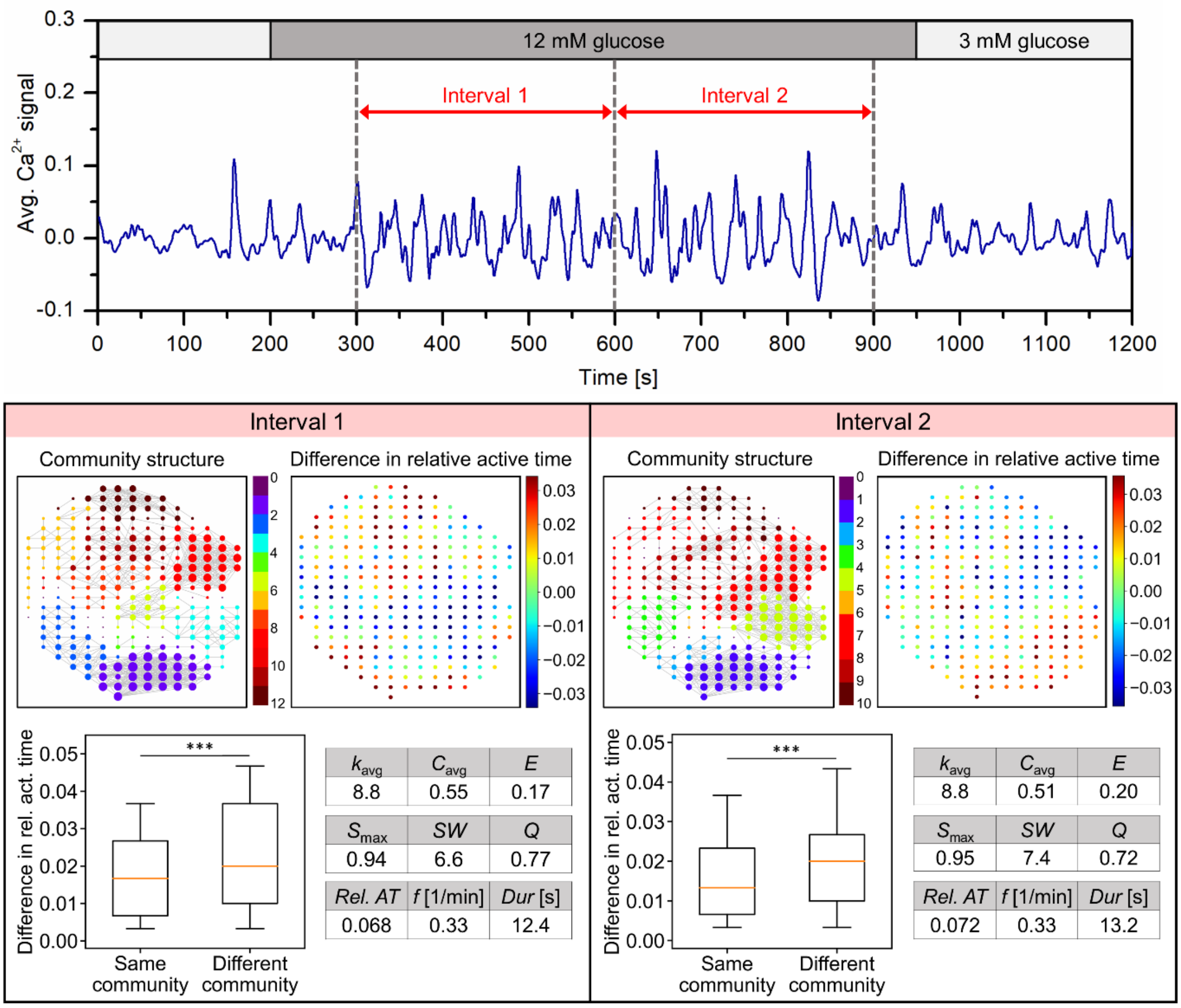

Figure S4: Temporal persistency of spatial distribution of functional heterogeneity in beta cell networks in a human islet. The upper panel shows the average Ca^{2+} signal from all cells within the islet, stimulated with 12 mM glucose, with designated time intervals used for network analysis. The lower panel is divided into Interval 1 and Interval 2, which were used for further analysis. For each time interval, functional beta cell networks were constructed. In these networks, colored dots represent the physical locations of islet subregions within the islet, grey lines indicate functional connections, and the colors of the subregions denote different communities. Next to this, islet subregions within the islet are color-coded according to their differences in relative active time, as specified by the color bar. Individual values are defined as the difference between the active time of a single islet subregion and the average active time of the entire islet. Additionally, the differences in active time within the same and different communities are shown for both intervals. Tables on the right summarize the calculated network parameters: average node degree (k_{avg}), average clustering coefficient (C_{avg}), global efficiency (*E)*, relative size of the largest component (S_{max}), small-world coefficient (*SW*), and modularity (*Q*). It also includes key cell activity parameters: relative active time (*AT*), average oscillation frequency (*f*), and average oscillation duration (*Dur*). Statistical significance: $*p < 0.05$, $**p < 0.01$, $***p < 0.001$; n.s., not significant.
